# Supplementary material for: Genome-wide Comparative Analysis of Atopic Dermatitis and Psoriasis Gives Insight into Opposing Genetic Mechanisms
Source: Am J Hum Genet. 2015 Jan 8;96(1):104–20. doi: 10.1016/j.ajhg.2014.12.004 (PMC4289690; doi:10.1016/j.ajhg.2014.12.004)
Supplement: Document S1. Supplemental Consortia Information, Figures S1–S6, and Tables S1–S4 and S6 [file mmc1.pdf]

The American Journal of Human Genetics
Supplemental Data

Genome-wide Comparative Analysis of Atopic Dermatitis and Psoriasis Gives Insight into Opposing Genetic Mechanisms

Hansjörg Baurecht, Melanie Hotze, Stephan Brand, Carsten Büning, Paul Cormican, Aiden Corvin, David Ellinghaus, Eva Ellinghaus, Jorge Esparza-Gordillo, Regina Fölster-Holst, Andre Franke, Christian Gieger, Norbert Hubner, Thomas Illig, Alan D. Irvine, Michael Kabesch, Young A.E. Lee, Wolfgang Lieb, Ingo Marenholz, W.H. Irwin McLean, Derek W. Morris, Ulrich Mrowietz, Rajan Nair, Markus M. Nöthen, Natalija Novak, Grainne M. O'Regan, the PAGE Consortium, Stefan Schreiber, Catherine Smith, Konstantin Strauch, Philip E. Stuart, Richard Trembath, Lam C. Tsoi, Michael Weichenthal, Jonathan Barker, James T. Elder, Stephan Weidinger, Heather J. Cordell, and Sara J. Brown

Supplemental Consortia Information

Membership of the PAGE (Population Architecture using Genomics and Epidemiology) consortium

University of Michigan (UMich)

Trilokraj Tejasvi,[1] Johann E. Gudjonsson,[1] John J Voorhees,[1] Jun Ding,[2] Yanming Li,[2] Hyun M Kang,[2] Goncalo R Abecasis[2]

1 Department of Dermatology, University of Michigan Ann Arbor, MI 48109, USA

2 Department of Biostatistics, Center for Statistical Genetics, University of Michigan Ann Arbor, MI 48109, USA

University of Toronto (UToronto)

Dafna D Gladman,[3] Fawnda J Pellett,[3] Vinod Chandran,[3] Cheryl F Rosen[4]

3 Department of Medicine, Division of Rheumatology, University of Toronto, Toronto Western Hospital, Toronto, Ontario M5T 2S8, Canada

4 Department of Medicine, Division of Dermatology, University of Toronto, Toronto Western Hospital, Toronto, Ontario MST2S8, Canada

Memorial University (MU)

Proton Rahman[5]

5 Department of Medicine, Memorial University, St. John's, Newfoundland A1C 5B8, Canada

University of Tartu (UTartu) and Estonian Genome Center Univeristy of Tartu (EGCUT)

Sulev Koks,[6] Külli Kingo,[7] Tonu Esko,[8] Andres Metspalu[8]

6 Department of Physiology, Centre of Translational Medicine and Centre for Translational Genomics, University of Tartu, 50409 Tartu, Estonia

7 Department of Dermatology and Venerology, University of Tartu, 50409 Tartu, Estonia

8 Estonian Genome Center and Center of Translational Genomics; Estonian Biocenter; Institute of Molecular and Cell Biology, University of Tartu, 50409 Tartu, Estonia

The Feinstein Institute for Medical Research (FIMR)

Peter Gregersen[9]

9 Robert S. Boas Center for Genomics and Human Genetics, The Feinstein Institute for Medical Research, Manhasset, NY 11030

National Psoriasis Victor Henschel BioBank (NPF)

Andrew Henschel,[10] Marin Aurand,[10] Bruce Bebo[10]

10 National Psoriasis Foundation, Portland, OR 97223 USA;

Henry Ford Hospital (HFH)

Henry W Lim[11]

11 Henry Ford Hospital, Detroit, Michigan, 48202, USA

Supplemental Figures

Figure S1. CCMA calibration curve

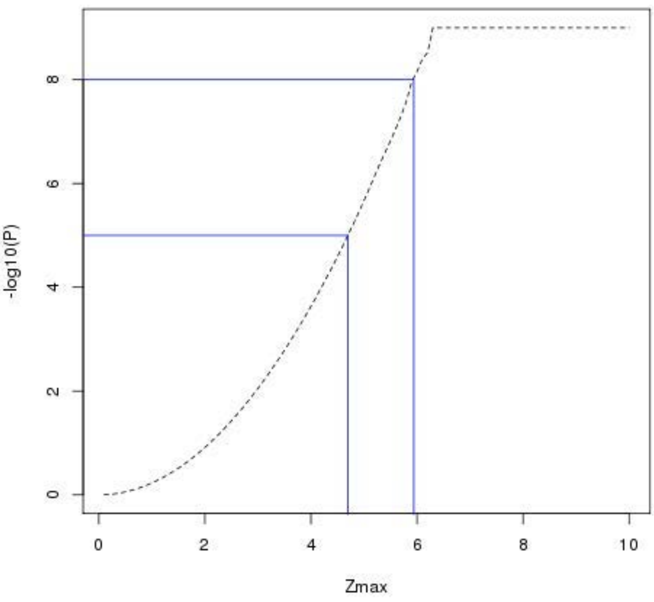

Calibration curve of the Z_{max} statistic based on 10^9 simulation replicates. Thresholds of Z_{max}=4.7 and Z_{max}=6.0 correspond to approximate genome-wide "suggestive" and "significant" thresholds ($P=1x10^{-5}$ and $1x10^{-8}$) respectively.

Figure S2. Mirrored Manhattan Plot of CCMA (upper panel) and MANTRA analysis (lower panel)

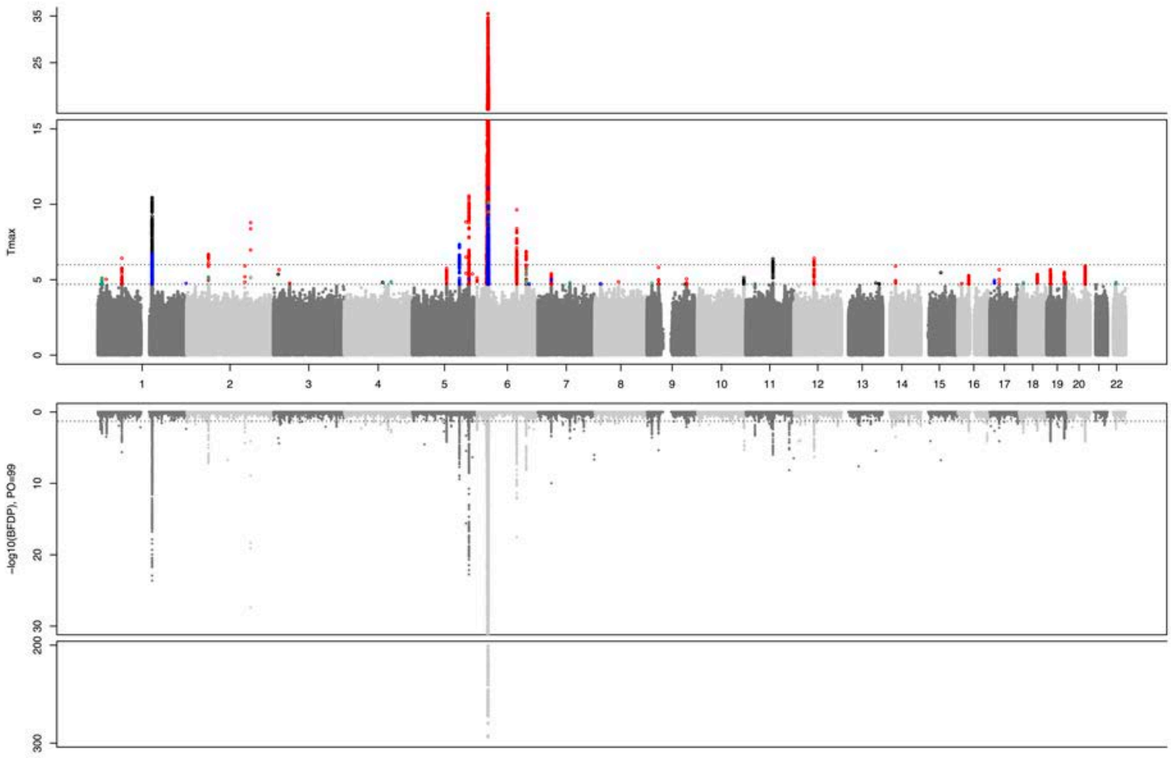

In the case-control meta-analysis (CCMA) the SNVs are color-coded as follows: **red** indicates psoriasis-specific; **black** AD-specific; **blue** opposing effect; **green** shared effect; the genome-wide significance threshold is indicated at T_{max}=6.0 (suggestive significance at T_{max}=4.7) for CCMA and Bayesian False Discovery Probability BFDP=0.05 for MANTRA.

Figure S3. Agreement of CCMA and MNM effect classification

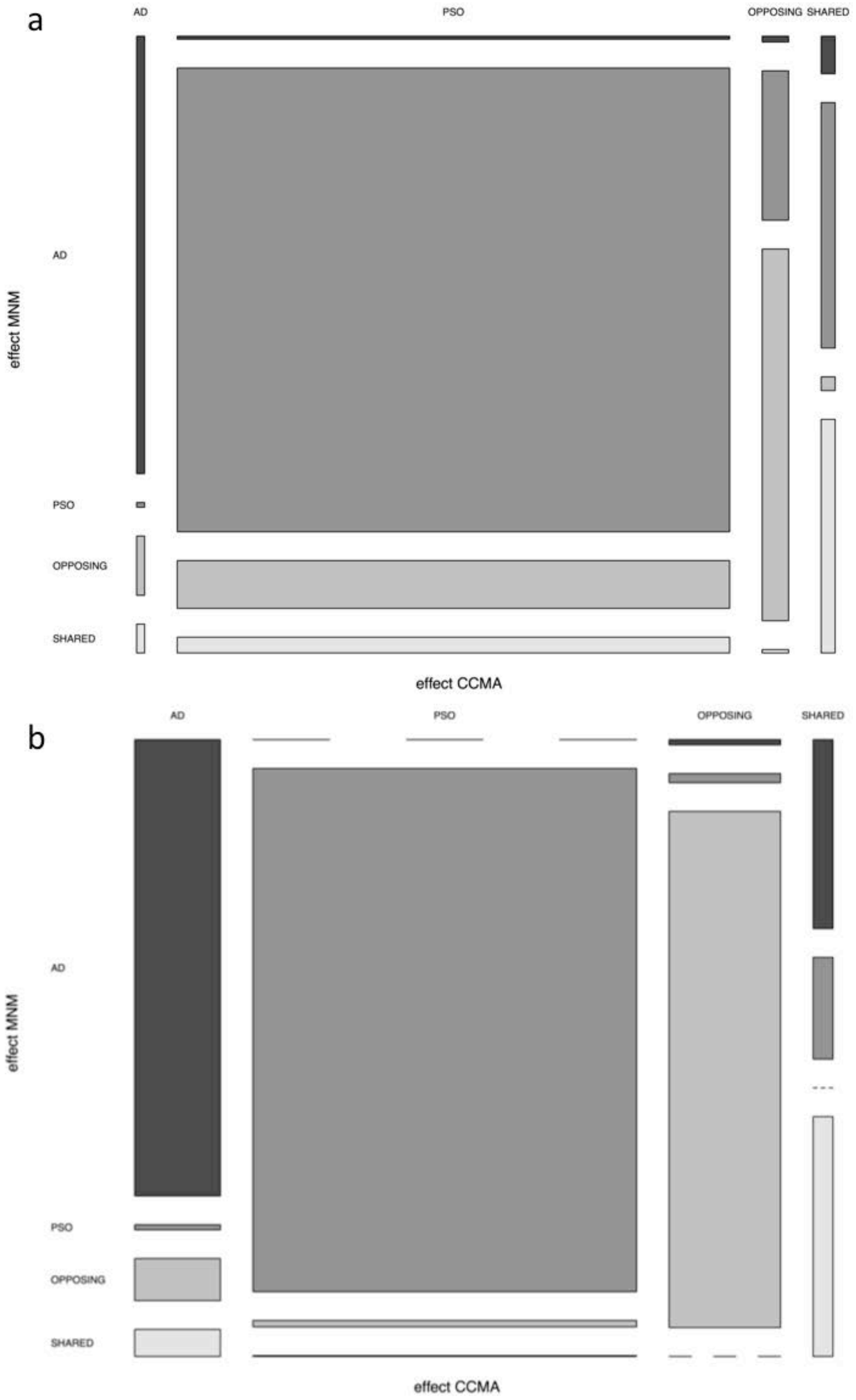

Mosaic plot showing the agreement of SNV effect classification into AD, psoriasis (PSO), shared and opposing effects for filtered SNVs (T_{max}>4.7) using CCMA and the multinomial models (MNM); (a) genome-wide comparison of both methods revealed 85.6% agreement, and (b) excluding the complex HLA region the comparison showed 94.8% agreement.

Figure S4: Association Plot and LD Structure of the EDC (left) and 5q31.1 (right)

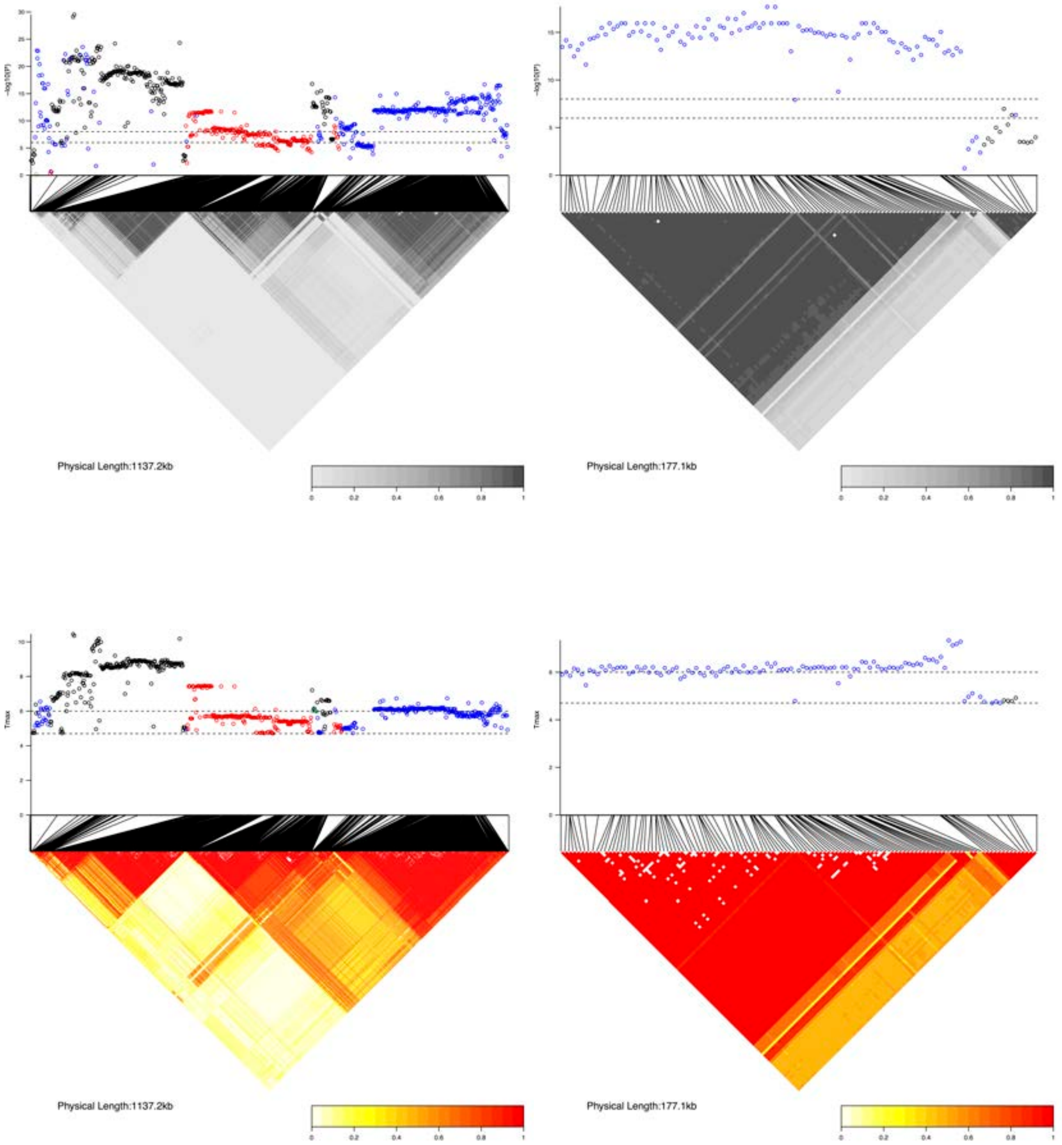

The upper plots display -$\log_{10}$(P) from the multinomial regression model and R^2 LD values; the lower plots display T_{max} from the CCMA and D' LD values; dashed lines indicate suggestive and genome-wide significance thresholds; color codes: **red**=psoriasis, **black**=AD, and **blue**=opposing effect.

Figure S5: Association Plot and LD Structure of the cytokine cluster at 5q31.1 using Immunochip data

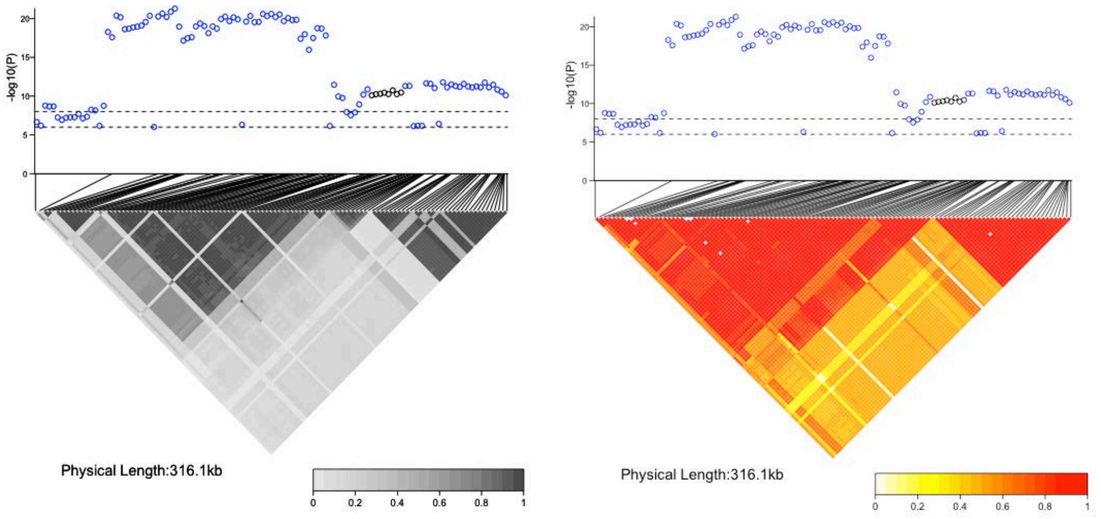

R^2 (left) and D’ (right) together with -$\log_{10}$(P) from the multinomial regression model are displayed; color codes: **black**=AD-specific effect and **blue**=opposing effect on AD and psoriasis.

Figure S6: Predicted protein network and gene ontology analyses

(a) 32 protein products & interactions predicted from 33 genes showing significance in genome-wide and/or conditional analyses

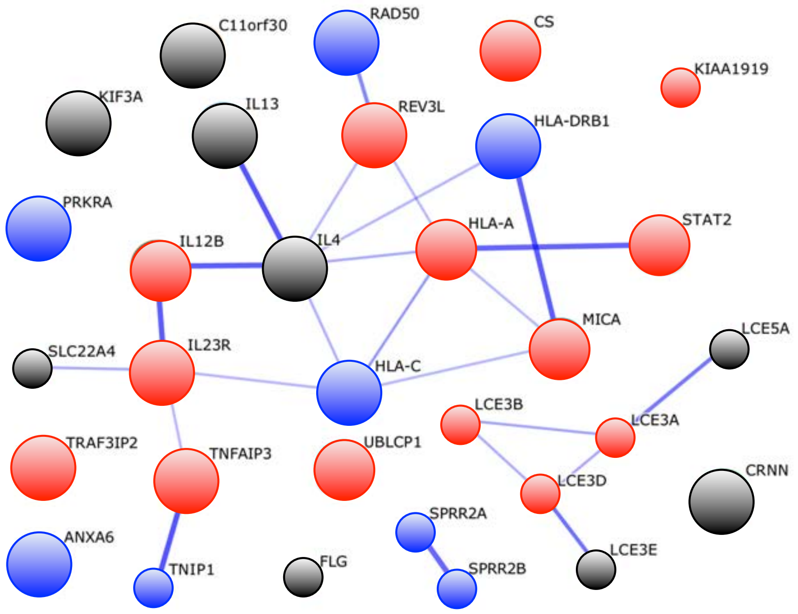

Colors indicate **AD-specific,** **psoriasis-specific** and **AD-psoriasis opposing** effects

GO:0031424	keratinization	n=7	p=3.08E-08
GO:0030216	keratinocyte differentiation	n=8	p=3.08E-08
GO:0009913	epidermal cell differentiation	n=8	p=2.64E-07
GO:0008544	epidermis development	n=8	p=4.90E-05
GO:0043588	skin development	n=8	p=1.11E-04
GO:2000319	regulation of T-helper 17 cell differentiation	n=3	p=1.50E-04
GO:2000316	regulation of T-helper 17 type immune response	n=3	p=2.57E-04

(b) 17 protein products & interactions predicted from 18 genes showing significant association with AD (AD-specific and opposing)

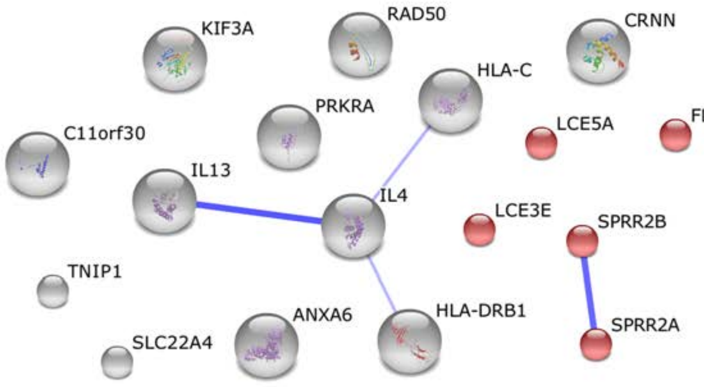

GO:0030216	**keratinocyte differentiation**	**n=5 marked red**	p=4.30E-04
GO:0031424	keratinization	n=4	p=6.94E-04
GO:0009913	epidermal cell differentiation	n=5	p=6.94E-04
GO:0008544	epidermis development	n=5	p=1.60E-02
GO:0043588	skin development	n=5	p=2.46E-02

(c) 23 protein products & interactions predicted from 24 genes showing significant association with psoriasis (psoriasis-specific and opposing)

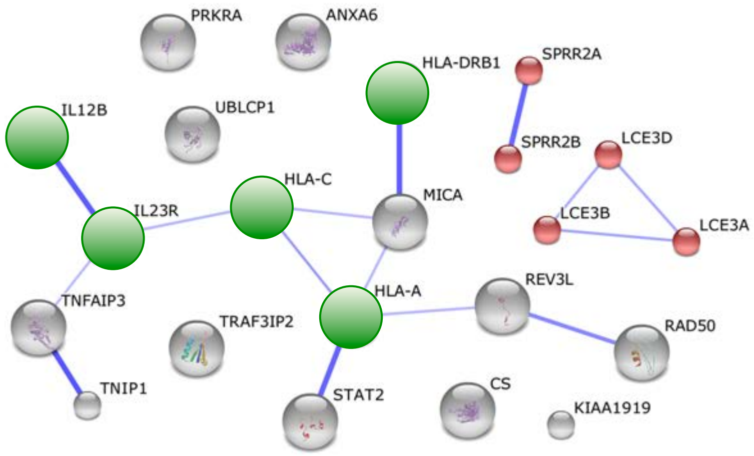

GO:0031424	**keratinization**	**n=5 marked red**	p=4.77E-05
GO:0030216	**keratinocyte differentiation**	**n=5 marked red**	p=6.94E-04
GO:0009913	epidermal cell differentiation	n=5	p=1.89E-03
GO:0034341	**response to interferon-gamma**	**n=5 marked green**	p=1.89E-03
GO:0050688	regulation of defense response to virus	n=4	p=8.11E-03

(d) 9 protein products from 12 genes showing opposing actions in AD and psoriasis

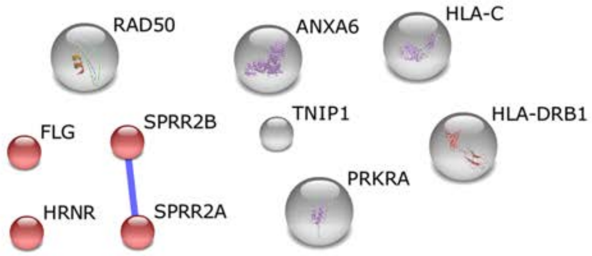

GO:0030216	**keratinocyte differentiation**	**n=4 marked red**	p=2.70E-03
GO:0009913	**epidermal cell differentiation**	**n=4 marked red**	p=4.78E-03
GO:0031424	keratinization	n=3	p=1.24E-02
GO:0061436	establishment of skin barrier	n=2	p=3.02E-02
GO:0008544	epidermis development	n=4	p=3.02E-02
GO:0033561	regulation of water loss via skin	n=2	p=4.55E-02

Genes identified from the results of analyses presented in Tables 1, 2 and 3; $STRING_{9.1}$ http://string-db.org/ accessed 17 July 2014; these are confidence views in which stronger associations are represented by thicker lines; n=number of genes; p values are corrected by FDR.

Supplemental Tables

Table S1. Case and control collections for AD and psoriasis

Panel	Numbers		Collection	Platform	Previous publications
**GWAS data**					
A	AD	663	Munich/ Bonn	Illumina 300k	[1]
Germany	Controls	786	PopGen/ ISAAC		
B	AD	993	Munich/ Kiel/ Berlin	Affymetrix 6.0	[2]
Germany	Controls	1513	KORA		
C	AD	606	Dublin/ Dundee	Illumina 610k	[1; 3]
Ireland	Controls	1794	TRINITY (Dublin)	Affymetrix 6.0	
D	Psoriasis	492	Kiel	Illlumina 550k	[4]
Germany	Controls	1161	PopGen/ KORA		
E	Psoriasis	2622	WTCCC2	Illumina 1M	[5-7]
United Kingdom	Controls	5667			
F	Psoriasis	1375	CASP	Perlegen	[8]
United States	Controls	1412			
**Immunochip data**					
Germany	AD	2425	Munich/ Bonn/ Berlin	Immunochip	[3; 5]
	Psoriasis	572	Kiel		
	Controls	5449	PopGen/ KORA/ HNR/ Munich/ Berlin/ Bonn		
United States	Psoriasis	1351	UMich/NPH/HFH		
	Controls	2694	UMich/FIMR/NPH		
Canada	Psoriasis	362	UToronto/MU		
	Controls	20	UToronto		
Estonia	Psoriasis	1295	UTartu/ EGCUT		
	Controls	898	EGCUT		

PopGen, PopGen biorepository; ISAAC, International Study of Asthma and Allergies in Childhood; TRINITY, Trinity Biobank Controls, Dublin, Ireland; WTCCC2, Wellcome Trust Case Control Consortium 2; HNR, Heinz Nixdorf Recall study; CASP, Collaborative Association Study of Psoriasis; UMich, University of Michigan; HFH, Henry Ford Hospital, NPF, National Psoriasis Foundation Victor Henschel BioBank; FIMR, The Feinstein Institute for Medical Research; UToronto, University of Toronto; MU, Memorial Hospital Newfoundland; UTartu, University of Tartu; EGCUT, Estonian Genome Center University of Tartu.

Table S2. Summary data before imputation

Data set	# Individuals	Cases	Controls	# SNPs before QC	# SNPs after QC
German AD Illumina	1,381	615	766	275,099	264,658
German AD Affymetrix	2,196	892	1,304	783,889	481,680
Irish AD Illumina/Affymetrix	2,369	572	1,797	143,001	131,629
German Psoriasis	1,598	475	1,123	561,466	493,992
UK Psoriasis	7,893	2,382	5,511	542,011	508,084
US Psoriasis	2,753	1,355	1,398	599,164	327,097
	18,190	6,291	11,834		

Table S3. Summary data after imputation

Cohort	#SNPs after imputation	# SNPs failed imputation QC	# SNPs after imputation QC
German AD Illumina	5,513,905	84,450	5,429,455
German AD Affymetrix	5,552,779	75,913	5,476,866
Irish AD Illumina/Affymetrix	5,489,685	353,166	5,136,519
German Psoriasis	5,559,007	76,170	5,482,837
UK Psoriasis	5,562,502	69,402	5,493,100
US Psoriasis	5,534,690	79,330	5,455,360

Table S4. Components of the prior matrix for MANTRA

$D_{Ethnicity}$

	gerA	gerI	Ire	gerP	UK	US
gerA	0					
gerI	0.0021	0				
Ire	0.0194	0.0194	0			
gerP	0.0033	0.0024	0.0191	0		
UK	0.0109	0.0115	0.0105	0.0107	0	
US	0.0061	0.0072	0.0143	0.0072	0.0053	0

$D_{Disease}$

	gerA	gerI	Ire	gerP	UK	US
gerA	0					
gerI	0	0				
Ire	0.02	0.02	0			
gerP	0.04	0.04	0.04	0		
UK	0.04	0.04	0.04	0	0	
US	0.04	0.04	0.04	0	0	0

D_{Total}

	gerA	gerI	Ire	gerP	UK	US
gerA	0					
gerI	0.0021	0				
Ire	0.0394	0.0394	0			
gerP	0.0433	0.0424	0.0591	0		
UK	0.0509	0.0515	0.0505	0.0107	0	
US	0.0461	0.0472	0.0543	0.0072	0.0053	0

The disease prior for the MANTRA method was specified by two components (a) $D_{Ethnicity}$ showing the Euclidean distances between study centers based on the genetic distance derived by the MDS analysis of the IBS matrix and (b) $D_{Disease}$ an arbitrary distance set to distinguish both diseases (see **Methods** section). gerA: German Affymentrix AD cohort (Panel B); gerA: German Illumina AD cohort (Panel A); Ire: Irish AD cohort (Panel C); gerP: German psoriasis cohort (Panel D); UK: UK psoriasis cohort (Panel E); US: US psoriasis cohort (Panel F).

Table S5. Results of CCMA and MNM analyses showing significance levels and comparison with known susceptibility loci for AD and psoriasis

[Supplemental Table 5 provided as Excel file]

Lead SNP defined by clumping procedure (Purcell, S. et al. PLINK: a tool set for whole-genome association and population-based linkage analyses. Am J Hum Genet 81, 559-75 2007); genome-wide significant results defined as Tmax>6, BFDP<0.05 with PO=1/999 or PMNM<10^{-8} are marked in bold; genes and transcripts identified from UCSC Genome Browser Human Feb. 2009 (GRCh37/hg19) Assembly accessed 21 March 2014.

Table S6. Summary of previous GWAS findings and comparison with our findings

S6(a) Atopic Dermatitis

Locus	Reported gene(s)	Reported SNV(s)	Associated SNV in region (+/-250kb)	Position	EA / RA	OR (95% CI)	P-value	Reference	SNV represented in disease-specific/ comparative analyses?
1q21.3	*tags FLG signal*	rs3126085	rs11205006	152440176	A/T	1.615(1.543-1.691)	1.57E-25	[2; 9]	913/280
2q12.1	*IL18R1, IL18RAP ,SLC9A4*	rs13015714[a] rs759382	rs759382	103094213	T/G	0.857(0.815-0.9)	0.0017	[3; 10]	1361/0
4q27	*IL2, IL21*	rs17389644	rs17389644	123497697	A/G	1.208(1.119–1.304)	1.16E−06	[3]	550/13
5q31.1	*RAD50, IL13, IL4, KIF3A*	rs1295686[a] rs2897442	rs1295686	131995843	T/C	1.347(1.28-1.418)	5.30E-09	[10; 11]	765/121
6p21.33	*HLA-C, HLA-B, MICA*	rs9368677[a] rs2251396	rs148203517	31324100	T/G	1.305(1.239-1.375)	2.95E-07	[1; 10]	8172/6065
6p21.33	*BAT1*	rs2844509	rs2844509	31510924	A/G	1.211(1.097-1.337)	1.50E-04	[1]	5429/3596
6p21.33	*C6orf48*	rs9368699	rs9368699	31802541	C/T	0.657(0.534-0.809)	7.78E-05	[1]	719/366
6p21.33	*TNXB, CREBL1*	rs12153855	rs12153855	32074804	C/T	0.781(0.679-0.898)	5.19E-04	[1]	1755/1027
11p12	*PRR5L*	rs12295535	rs12295535	36432024	T/C	1.382(1.202-1.589)	0.0204	[3]	1141/1
11q13.1	*OVOL1*	rs479844	rs479844	65551957	A/G	0.851(0.815-0.889)	2.06E-04	[10,11]	838/0
11q13.5	*C11orf30, LRRC32*	rs7927894, rs11236809[a]	rs7927894	76301316	T/C	1.265(1.21-1.322)	1.24E-07	[2; 10]	1104/48
16p13.13	*CLEC16A*	rs9923856[a] rs2041733	rs2041733	11229589	T/C	1.172(1.124-1.222)	1.51E-04	[3; 10]	1292/2
17q21.32 -33	*ZNF652*	rs16948048	rs16948048	47440466	G/A	1.101(1.012-1.198)	0.0252	[3]	991/0
19p13.2	*ACTL9*	rs2164983	rs2164983	8789381	A/C	1.129(0.999-1.275)	0.0521	[11]	1002/0
20q13.33	*TNFRSF6B*	rs909341	rs909341	62328742	G/A	1.316(1.205-1.429)	7.73E−10	[3]	1226/0
**Loci reported in Asian populations**									
3p22.3	*GLB1*	rs7613051	rs1607463	32910192	A/T	1.471(1.294-1.673)	0.0027	[10]	1032/0
3q13.2	*CCDC80*	rs12634229	rs58161637	112545910	G/GT	1.214(1.16-1.27)	2.21E-05	[10]	1038/0
5q22.1	*TMEM232, SLC25A46*	rs7701890	rs4957919	110100063	T/C	1.195(1.117-1.28)	0.0089	[9]	909/0
6p21.32	*GPSM3*	rs176095	rs176095	32158319	G/A	0.768(0.694-0.85)	3.44E-07	[10]	2622/1554
6p21.32	*C6orf10*	rs9469099	rs41268896	32070069	A/G	1.298(1.238-1.361)	4.16E-08	[10]	8274/5295
7p22.2	*CARD11*	rs4722404	rs6953573	3081727	A/G	1.194(1.136-1.255)	3.61E-04	[10]	1813/0
10q21.2	*ZNF365*	rs10995251	rs2393903	64380336	T/C	0.831(0.795-0.869)	3.42E-05	[10]	951/0
11p15.4	*OR10A3, NLRP10*	rs878860	rs4758289	8115699	T/C	1.163(1.099-1.231)	0.0079	[10]	1752/0
20q13.2	*CYP24A1, PFDN4*	rs16999165	rs176383	52613413	T/C	1.356(1.253-1.468)	1.19E-04	[10]	1286/0

EA/RA=effect allele/reference allele; [a]indicate reported Asian SNVs.

S6(b) Psoriasis

Locus	Reported gene(s)	Reported SNV(s)	Associated SNV in region (+/-250kb)	Position	EA / RA	OR (95% CI)	P-value	Reference	SNV represented in disease-specific/ comparative analyses?
1p36.23	*SLC45A1, TNFRSF9*	rs11121129	rs11121129	8268095	A/G	1.131(1.096-1.167)	8.74E-05	[5]	884/25
1p36.13	*CAPZB*	rs7667	rs7667	19718824	A/G	1.079(1.046-1.112)	0.0131	[4]	1127/0
1p36.11	*IL28RA*	rs4649203	rs4649203	24519920	A/G	1.153(1.113-1.195)	5.81E-05	[5; 7]	973/2
1p36.11	*RUNX3*	rs7536201	rs7536201	25293084	T/C	0.883(0.858-0.91)	2.37E-05	[5]	1051/50
1p31.3	*IL23R*	rs2201841, rs11209026 rs9988642	rs11209026	67705958	A/G	0.646(0.598-0.699)	2.90E-08	[5; 7; 8]	1066/60
1q21.3	*LCE3A, LCE3B, LCE3D*	rs4085613[a] rs4112788 rs6677595	rs4112788	152551276	A/G	0.791(0.766-0.816)	8.37E-14	[5; 7; 12]	1028/492
2p16.1	*FLJ16341, REL*	rs702873, rs62149416	rs702873	61081542	T/C	0.822(0.798-0.847)	3.22E-11	[5; 7; 13]	649/64
2p15	*B3GNT2*	rs10865331	rs10865331	62551472	A/G	1.131(1.067-1.198)	3.43E-05	[5]	836/0
2q24.2	*IFIH1*	rs17716942	rs17716942	163260691	T/C	1.309(1.198-1.433)	3.26E-09	[7]	459/3
3p24.3	*Intergenic*	rs6809854	rs6809854	18784423	A/G	0.885(0.854-0.917)	5.73E-04	[7]	1126/12
3q13.31	*ZDHHC23*	rs1386478	rs1386478	113680951	A/G	1.09(1.022-1.162)	0.00894	[4]	711/0
5q15	*ERAP1*	rs27524, rs27432	rs27432	96119273	A/G	1.195(1.157-1.234)	2.37E-08	[5; 7]	1199/65
5q31.1	*IL13, IL4*	rs20541, rs1295685	rs20541	131995964	A/G	0.841(0.81-0.874)	5.39E-06	[5; 8]	766/121
5q33.1	*ANXA6, TNIP1*	rs2233278, rs17728338	rs17728338	150478318	A/G	1.647(1.557-1.743)	1.07E-18	[5; 8]	1435/3
5q33.3	*IL12B*	rs2082412 rs3213094[a] rs2546890 rs12188300	rs2546890	158759900	A/G	1.368(1.328-1.41)	5.39E-26	[5; 8; 12; 14]	801/218
6p25.3	*EXOC2, IRF4*	rs9504361	rs9504361	577820	A/G	1.163(1.129-1.198)	2.96E-07	[5]	1240/15
6p21.33	*HLA-C*	rs1265181[a] rs12191877 rs4406273 rs10484554 rs3134792 rs2395029	rs2395029	31431780	G/T	5.00(4.695-5.348)	6.84E-138	[5; 8; 12; 15; 16]	6809/4811
6q21	*REV3L*	rs465969	rs465969	111655530	A/G	1.403(1.335-1.474)	7.82E-12	[7]	519/226
6q21	*TRAF3IP2*	rs240993, rs33980500	rs240993	111673714	T/C	1.282(1.241-1.324)	1.20E-14	[7; 14]	543/235
6q23.3	*TNFAIP3*	rs582757 rs610604	rs582757	138197824	T/C	0.802(0.777-0.828)	5.70E-12	[5; 7]	921/60
6q25.3	TAGAP	rs2451258	rs2451258	159506600	T/C	0.898(0.871-0.926)	4.13E-04	[5]	1558/4
7p14.1-2	*ELMO1*	rs2700987	rs2700987	37386237	A/C	1.174(1.14-1.209)	6.60E-08	[5]	1193/27
9p21.1	*DDX58*	rs11795343	rs11795343	32523737	T/C	1.19(1.155-1.226)	5.78E-09	[5]	893/29
9q31.2	*KLF4*	rs10979182	rs10979182	110817020	A/G	1.149(1.084-1.218)	2.99E-06	[5]	1265/6
9q34.13	*TSC1*	rs1076160	rs1076160	135776034	T/C	1.046(0.988-1.107)	0.1223	[8]	1079/0
10q22.3	*ZMIZ1*	rs1250546 rs1250544	rs1250546	81032532	A/G	1.114(1.05-1.182)	3.55E-04	[4; 5]	1197/0
11q13.1	*RPS6KA4, PRDX5*	rs645078	rs645078	64135298	A/C	1.095(1.032-1.163)	0.0029	[5]	608/0
11q22.3	*ZC3H12C*	rs4561177	rs4561177	109962432	A/G	1.122(1.059-1.189)	9.03E-05	[5]	858/0
11.q24.3	*ETS1*	rs3802826	rs3802826	128406438	A/G	1.116(1.054-1.182)	1.67E-04	[5]	1010/9
12q13.2	*RPS26*	rs12580100	rs12580100	56439209	A/G	1.284(1.171-1.408)	1.01E-07	[13]	315/40
12q13.3	*IL23A, STAT2*	rs2066808 rs2066819	rs2066808	56737973	A/G	1.477(1.305-1.671)	6.45E-10	[5; 8]	420/32
13q14.11	*COG6*	rs7993214	rs7993214	40350912	T/C	0.897(0.87-0.925)	3.66E-04	[16]	1133/0
14q13.2	*NFKBIA*	rs12586317 rs8016947	rs8016947	35832666	T/G	0.84(0.815-0.865)	3.90E-09	[7; 13]	916/8
16p11.2	*FBXL19, STX1B*	rs10782001 rs12445568	rs12445568	31004812	C/T	1.167(1.101-1.238)	2.65E-07	[5; 13]	343/100

S6(b) continued

Locus	Reported gene(s)	Reported SNV(s)	Associated SNV in region (+/-250kb)	Position	EA / RA	OR (95% CI)	P-value	Reference	SNV represented in disease-specific/ comparative analyses?
16p13.13	*PRM3, SOCS1*	rs4780355	rs4780355	11347858	T/C	1.165(1.127-1.203)	3.13E-06	[4]	1655/2
17q11.2	*NOS2*	rs4795067 rs28998802	rs4795067	26106675	A/G	0.843(0.818-0.869)	1.47E-08	[5; 13]	836/3
17q21.2	*PTRF, STAT3*	rs963986	rs963986	40561579	C/G	1.158(1.069-1.255)	3.21E-04	[5]	642/1
17q25.3	*CARD14*	rs11652075	rs11652075	78178893	T/C	0.885(0.86-0.912)	3.64E-05	[5]	1347/11
18q21.2	*POL1, STARD6, MBD2*	rs545979	rs545979	51819750	T/C	1.172(1.136-1.209)	3.29E-07	[5]	736/84
19p13.2	*TYK2*	rs34536443 rs12720356 rs280519	rs34536443	10463118	G/C	1.76(1.46-2.10)	3.4E-10	[5; 7]	902/29
19p13.2	*ILF3, CARM1*	rs892085	rs892085	10818092	A/G	1.172(1.103-1.245)	3.13E-07	[5]	810/42
20q13.12	*SDC4*	rs1008953	rs1008953	43980726	C/T	1.121(1.046-1.202)	0.0013	[13]	1351/0
20q13.13	*SPATA2, RNF114*	rs495337 rs1056198	rs495337	48522330	A/G	0.839(0.814-0.864)	3.43E-09	[5; 15]	1078/215
22q11.21	*UBE2L3*	rs4821124	rs4821124	21979289	C/T	1.159(1.076-1.248)	9.08E-05	[5]	535/0
**Loci reported in Asian populations**									
4q24	*NFKB1*	rs1020760 rs1609798	rs230503	103492669	A/G	0.923(0.893-0.955)	0.0170	[17]	835/0
5q33.3	*PTTG1*	rs2431697	rs2961918	159901408	A/C	1.111(1.075-1.149)	0.0015	[18]	1039/0
8p23.2	*CSMD1*	rs7007032	rs11136687	3809504	A/T	1.127(1.087-1.168)	7.88E-04	[18]	3475/0
12p13.3	Multiple genes: *CD27-LAG3*	rs758739 rs2243750	rs111922468	6556078	T/C	1.121(1.08-1.163)	0.0020	[17]	993/0
13q12.11	*GJB2*	rs3751385	rs7324150	20698681	A/G	0.878(0.84-0.917)	0.0029	[18]	1133/0
17q12	*IKZF3*	rs10852936 rs12936231	rs146489775	38185192	T/C	1.773(1.418-2.216)	0.0103	[17]	634/0
18q22.1	*SERPINB8*	rs514315	rs191358722	61730218	T/C	1.676(1.463-1.921)	1.51E-04	[18]	1215/0
19q13.33	*FUT2*	rs1047781	rs629504	49223633	C/G	0.845(0.819-0.871)	4.14E-08	[19]	1186/27
19q13.41	*ZNF816A*	rs12459008 rs9304742	rs12459008	53454789	A/T	1.162(1.090-1.238)	3.8E-06	[18; 19]	2016/13

EA/RA=effect allele/reference allele; [a]indicate reported Asian SNVs.

Table S7. Gene expression data in psoriasis and AD relating to candidate genes identified by comparative analysis

[Supplemental Table 7 provided as Excel file]

Gene expression data available from published and publically available datasets are summarized for candidate genes.

References

1. Weidinger, S., Willis-Owen, S.A., Kamatani, Y., Baurecht, H., Morar, N., Liang, L., Edser, P., Street, T., Rodriguez, E., O'Regan, G.M., et al. (2013). A genome-wide association study of atopic dermatitis identifies loci with overlapping effects on asthma and psoriasis. Hum Mol Genet 22, 4841-4856.

2. Esparza-Gordillo, J., Weidinger, S., Folster-Holst, R., Bauerfeind, A., Ruschendorf, F., Patone, G., Rohde, K., Marenholz, I., Schulz, F., Kerscher, T., et al. (2009). A common variant on chromosome 11q13 is associated with atopic dermatitis. Nat Genet 41, 596-601.

3. Ellinghaus, D., Baurecht, H., Esparza-Gordillo, J., Rodriguez, E., Matanovic, A., Marenholz, I., Hubner, N., Schaarschmidt, H., Novak, N., Michel, S., et al. (2013). High-density genotyping study identifies four new susceptibility loci for atopic dermatitis. Nature Genetics 45, 808-812.

4. Ellinghaus, D., Ellinghaus, E., Nair, R.P., Stuart, P.E., Esko, T., Metspalu, A., Debrus, S., Raelson, J.V., Tejasvi, T., Belouchi, M., et al. (2012). Combined analysis of genome-wide association studies for Crohn disease and psoriasis identifies seven shared susceptibility loci. American Journal of Human Genetics 90, 636-647.

5. Tsoi, L.C., Spain, S.L., Knight, J., Ellinghaus, E., Stuart, P.E., Capon, F., Ding, J., Li, Y., Tejasvi, T., Gudjonsson, J.E., et al. (2012). Identification of 15 new psoriasis susceptibility loci highlights the role of innate immunity. Nature Genetics 44, 1341-1348.

6. Knight, J., Spain, S.L., Capon, F., Hayday, A., Nestle, F.O., Clop, A., Wellcome Trust Case Control, C., Genetic Analysis of Psoriasis, C., Consortium, I.c.f.P., Barker, J.N., et al. (2012). Conditional analysis identifies three novel major histocompatibility complex loci associated with psoriasis. Hum Mol Genet 21, 5185-5192.

7. Genetic Analysis of Psoriasis Consortium, Wellcome Trust Case Control Consortium, Strange, A., Capon, F., Spencer, C.C., Knight, J., Weale, M.E., Allen, M.H., Barton, A., Band, G., et al. (2010). A genome-wide association study identifies new psoriasis susceptibility loci and an interaction between HLA-C and ERAP1. Nature Genetics 42, 985-990.

8. Nair, R.P., Duffin, K.C., Helms, C., Ding, J., Stuart, P.E., Goldgar, D., Gudjonsson, J.E., Li, Y., Tejasvi, T., Feng, B.J., et al. (2009). Genome-wide scan reveals association of psoriasis with IL-23 and NF-kappaB pathways. Nature Genetics 41, 199-204.

9. Sun, L.D., Xiao, F.L., Li, Y., Zhou, W.M., Tang, H.Y., Tang, X.F., Zhang, H., Schaarschmidt, H., Zuo, X.B., Foelster-Holst, R., et al. (2011). Genome-wide association study identifies two new susceptibility loci for atopic dermatitis in the Chinese Han population. Nat Genet 43, 690-694.

10. Hirota, T., Takahashi, A., Kubo, M., Tsunoda, T., Tomita, K., Sakashita, M., Yamada, T., Fujieda, S., Tanaka, S., Doi, S., et al. (2012). Genome-wide association study identifies eight new susceptibility loci for atopic dermatitis in the Japanese population. Nat Genet 44, 1222-1226.

11. Paternoster, L., Standl, M., Chen, C.M., Ramasamy, A., Bonnelykke, K., Duijts, L., Ferreira, M.A., Alves, A.C., Thyssen, J.P., Albrecht, E., et al. (2011). Meta-analysis of genome-wide association studies identifies three new risk loci for atopic dermatitis. Nature Genetics 44, 187-192.

12. Zhang, X.J., Huang, W., Yang, S., Sun, L.D., Zhang, F.Y., Zhu, Q.X., Zhang, F.R., Zhang, C., Du, W.H., Pu, X.M., et al. (2009). Psoriasis genome-wide association study identifies susceptibility variants within LCE gene cluster at 1q21. Nature Genetics 41, 205-210.

13. Stuart, P.E., Nair, R.P., Ellinghaus, E., Ding, J., Tejasvi, T., Gudjonsson, J.E., Li, Y., Weidinger, S., Eberlein, B., Gieger, C., et al. (2010). Genome-wide association analysis identifies three psoriasis susceptibility loci. Nature Genetics 42, 1000-1004.

14. Ellinghaus, E., Ellinghaus, D., Stuart, P.E., Nair, R.P., Debrus, S., Raelson, J.V., Belouchi, M., Fournier, H., Reinhard, C., Ding, J., et al. (2010). Genome-wide association study identifies a psoriasis susceptibility locus at TRAF3IP2. Nature Genetics 42, 991-995.

15. Capon, F., Bijlmakers, M.J., Wolf, N., Quaranta, M., Huffmeier, U., Allen, M., Timms, K., Abkevich, V., Gutin, A., Smith, R., et al. (2008). Identification of ZNF313/RNF114 as a novel psoriasis susceptibility gene. Human molecular genetics 17, 1938-1945.

16. Liu, Y., Helms, C., Liao, W., Zaba, L.C., Duan, S., Gardner, J., Wise, C., Miner, A., Malloy, M.J., Pullinger, C.R., et al. (2008). A genome-wide association study of psoriasis and psoriatic arthritis identifies new disease loci. PLoS Genet 4, e1000041.

17. Sheng, Y., Jin, X., Xu, J., Gao, J., Du, X., Duan, D., Li, B., Zhao, J., Zhan, W., Tang, H., et al. (2014). Sequencing-based approach identified three new susceptibility loci for psoriasis. Nature communications 5, 4331.

18. Sun, L.D., Cheng, H., Wang, Z.X., Zhang, A.P., Wang, P.G., Xu, J.H., Zhu, Q.X., Zhou, H.S., Ellinghaus, E., Zhang, F.R., et al. (2010). Association analyses identify six new psoriasis susceptibility loci in the Chinese population. Nature genetics 42, 1005-1009.

19. Tang, H., Jin, X., Li, Y., Jiang, H., Tang, X., Yang, X., Cheng, H., Qiu, Y., Chen, G., Mei, J., et al. (2014). A large-scale screen for coding variants predisposing to psoriasis. Nature genetics 46, 45-50.
