# Supplementary material for: Genome-wide Comparative Analysis of Atopic Dermatitis and Psoriasis Gives Insight into Opposing Genetic Mechanisms
Source: Am J Hum Genet. 2015 Jan 8;96(1):104–20. doi: 10.1016/j.ajhg.2014.12.004 (PMC4289690; doi:10.1016/j.ajhg.2014.12.004)
Supplement: Document S2. Article plus Supplemental Data [file mmc4.pdf]

ARTICLE

Genome-wide Comparative Analysis of Atopic Dermatitis and Psoriasis Gives Insight into Opposing Genetic Mechanisms

Hansjörg Baurecht,[1,31] Melanie Hotze,[1,31] Stephan Brand,[2] Carsten Büning,[3] Paul Cormican,[4] Aiden Corvin,[4] David Ellinghaus,[5] Eva Ellinghaus,[5] Jorge Esparza-Gordillo,[6,7] Regina Fölster-Holst,[1] Andre Franke,[5] Christian Gieger,[8,9,10] Norbert Hubner,[6] Thomas Illig,[9,11] Alan D. Irvine,[12,13,14] Michael Kabesch,[15] Young A.E. Lee,[6,7] Wolfgang Lieb,[16] Ingo Marenholz,[6,7] W.H. Irwin McLean,[17] Derek W. Morris,[4,18] Ulrich Mrowietz,[1] Rajan Nair,[19] Markus M. Nöthen,[20,21] Natalija Novak,[22] Grainne M. O'Regan,[12] the PAGE Consortium, Stefan Schreiber,[5,23] Catherine Smith,[24] Konstantin Strauch,[8,25] Philip E. Stuart,[19] Richard Trembath,[26] Lam C. Tsoi,[27] Michael Weichenthal,[1] Jonathan Barker,[24] James T. Elder,[19,28] Stephan Weidinger,[1,32,*] Heather J. Cordell,[29,32] and Sara J. Brown[30,32,*]

Atopic dermatitis and psoriasis are the two most common immune-mediated inflammatory disorders affecting the skin. Genome-wide studies demonstrate a high degree of genetic overlap, but these diseases have mutually exclusive clinical phenotypes and opposing immune mechanisms. Despite their prevalence, atopic dermatitis and psoriasis very rarely co-occur within one individual. By utilizing genome-wide association study and ImmunoChip data from >19,000 individuals and methodologies developed from meta-analysis, we have identified opposing risk alleles at shared loci as well as independent disease-specific loci within the epidermal differentiation complex (chromosome 1q21.3), the Th2 locus control region (chromosome 5q31.1), and the major histocompatibility complex (chromosome 6p21–22). We further identified previously unreported pleiotropic alleles with opposing effects on atopic dermatitis and psoriasis risk in *PRKRA* and *ANXA6/TNIP1*. In contrast, there was no evidence for shared loci with effects operating in the same direction on both diseases. Our results show that atopic dermatitis and psoriasis have distinct genetic mechanisms with opposing effects in shared pathways influencing epidermal differentiation and immune response. The statistical analysis methods developed in the conduct of this study have produced additional insight from previously published data sets. The approach is likely to be applicable to the investigation of the genetic basis of other complex traits with overlapping and distinct clinical features.

Introduction

Atopic dermatitis (AD, synonymous with eczema [MIM 603165]) and psoriasis (psoriasis vulgaris [MIM 177900]) are the two most common chronic inflammatory skin conditions. They are associated with a significantly reduced quality of life and multiple comorbidities.[1,2] Both diseases result from the interaction of genetic and environmental factors and are characterized by epidermal defects as well as local and systemic immunological abnormalities.

[1]Department of Dermatology, Allergology, and Venereology, University Hospital Schleswig-Holstein, Campus Kiel, 24105 Kiel, Germany; [2]Department of Medicine II - Grosshadern, Ludwig-Maximilians-University (LMU), 81377 Munich, Germany; [3]Department of Gastroenterology, Hepatology and Endocrinology, Charité, Campus Mitte, 10117 Berlin, Germany; [4]Neuropsychiatric Genetics Research Group, Department of Psychiatry and Institute of Molecular Medicine, Trinity College Dublin, Dublin 2, Ireland; [5]Institute of Clinical Molecular Biology, Christian-Albrechts-University of Kiel, 24105 Kiel, Germany; [6]Max-Delbrück-Centrum (MDC) for Molecular Medicine, Berlin-Buch, 13092 Berlin, Germany; [7]Pediatric Allergy, Experimental and Clinical Research Center, Charité Universitätsmedizin Berlin, 10117 Berlin, Germany; [8]Institute of Genetic Epidemiology, Helmholtz Zentrum München, German Research Center for Environmental Health, 85764 Neuherberg, Germany; [9]Research Unit of Molecular Epidemiology, German Research Center for Environmental Health, 85764 Neuherberg, Germany; [10]Institute of Epidemiology II, Helmholtz Zentrum München - German Research Center for Environmental Health, 85764 Neuherberg, Germany; [11]Hannover Unified Biobank, Hannover Medical School, 30625 Hannover, Germany; [12]Department of Paediatric Dermatology, Our Lady's Children's Hospital, Crumlin, Dublin 12, Ireland; [13]National Children's Research Centre, Dublin 12, Ireland; [14]Department of Clinical Medicine, Trinity College Dublin, Dublin 2, Ireland; [15]Department of Pediatric Pneumology and Allergy, University Children's Hospital Regensburg (KUNO), 93042 Regensburg, Germany; [16]Institute of Epidemiology and PopGen Biobank, University Hospital Schleswig-Holstein, 24105 Kiel, Germany; [17]Dermatology and Genetic Medicine, College of Life Sciences and College of Medicine, Dentistry & Nursing, University of Dundee, Dundee DD1 5EH, UK; [18]Cognitive Genetics and Therapy Group, School of Psychology and Discipline of Biochemistry, National University of Ireland, Galway, Ireland; [19]Department of Dermatology, University of Michigan, Ann Arbor, MI 48109-5675, USA; [20]Institute of Human Genetics, University of Bonn, 53127 Bonn, Germany; [21]Department of Genomics, Life & Brain Center, University of Bonn, 53127 Bonn, Germany; [22]Department of Dermatology and Allergy, University of Bonn Medical Center, 53105 Bonn, Germany; [23]Department of Internal Medicine, University Medical Center Schleswig-Holstein, 24105 Kiel, Germany; [24]Division of Genetics and Molecular Medicine, St John's Institute of Dermatology, Kings College London, London SE1 9RT, UK; [25]Institute of Medical Informatics, Biometry and Epidemiology, Chair of Genetic Epidemiology, Ludwig-Maximilians-Universität, 80539 Munich, Germany; [26]Queen Mary University of London, Barts and the London School of Medicine and Dentistry, London E1 4NS, UK; [27]Department of Biostatistics, Center for Statistical Genetics, University of Michigan School of Public Health, Ann Arbor, MI 48109-5314, USA; [28]Ann Arbor Veterans Affairs Hospital, Ann Arbor, MI 48105, USA; [29]Institute of Genetic Medicine, Newcastle University, Newcastle upon Tyne NE1 3BZ, UK; [30]Dermatology and Genetic Medicine, Medical Research Institute, Ninewells Hospital and Medical School, University of Dundee, Dundee DD1 9SY, UK
[31]These authors contributed equally to this work
[32]These authors contributed equally to this work
*Correspondence: sweidinger@dermatology.uni-kiel.de (S.W.), s.j.brown@dundee.ac.uk (S.J.B.)
http://dx.doi.org/10.1016/j.ajhg.2014.12.004. ©2015 The Authors
This is an open access article under the CC BY license (http://creativecommons.org/licenses/by/3.0/).

Despite a lifetime prevalence of ~2% for psoriasis and 10%–20% for AD,[3,4] these diseases rarely co-occur within an individual[5]—an observation attributed to opposing immune response patterns.[6] However, it has been reported that both Th1-cell-dominated autoimmune and Th2-cell-dominated allergic diseases aggregate within families[7] and that parental psoriasis might increase the risk of AD in offspring.[8] Furthermore, genome-wide linkage and association studies have shown genetic risk loci in each disease that map to similar regions of the genome. The epidermal differentiation complex (EDC) on chromosome 1q21.3 includes AD and psoriasis risk loci in close proximity.[9–12] Null mutations in the gene encoding filaggrin (*FLG* [MIM 135940]) represent the strongest known risk factor for AD[13,14] and account for at least a proportion of AD risk within the EDC, but *FLG*-null mutations are not associated with psoriasis.[15,16] A deletion of the late cornified envelope genes *LCE3B-LCE3C* (MIM 612614, 612615) represents a genetic substrate for psoriasis within the EDC,[17,18] but this deletion is not associated with AD.[19] The cytokine cluster encoded at 5q23.1–5q31.1 includes variants showing association with both diseases,[10,20,21] and an intergenic region of chromosome 20q13.2 has also shown association with both AD and psoriasis.[22,23] Finally, a recent genome-wide association study (GWAS) on AD identified a strong association within the margins of the major histocompatibility complex (MHC)[20] on chromosome 6p21.3, less than 2.4 kb from a variant associated with HLA-Cw6 (MIM 142840),[24] the strongest known psoriasis-risk locus.

In order to gain insight into overlapping and specific genetic mechanisms, we systematically compared and contrasted AD and psoriasis via analytical techniques developed from meta-analysis.

Subjects and Methods

Study Subjects

Genome-wide genotype data were obtained on samples from six case-control cohorts (three each of AD and psoriasis), totaling 2,262 AD and 4,489 psoriasis case subjects and 12,333 control subjects (Table S1 available online).

The German AD case subjects were recruited from tertiary dermatology clinics at Munich, as part of the GENEVA study, University of Kiel, University of Bonn, and the University Children's Hospital of Charité Universitätsmedizin Berlin. AD was diagnosed by experienced dermatologists and/or pediatricians according to the UK Diagnostic Criteria.[25] German control subjects were obtained from the PopGen biorepository,[26] the population-based KORA study in southern Germany,[27] and the German part of ISAAC II to assess the prevalence of asthma and allergies in schoolchildren.[28] The Irish AD case collection was recruited from the secondary and tertiary pediatric dermatology clinic at Our Lady's Children's Hospital, Crumlin, Dublin. Irish control individuals were obtained from healthy adult blood donors as part of the Trinity Biobank, Dublin.[29]

The German psoriasis case subjects were recruited from the tertiary dermatology clinic at the University of Kiel and German controls were again obtained from the PopGen biorepository and the KORA study (independent from those used as controls for AD). The British psoriasis case-control study is part of the Welcome Trust Case Control Consortium 2[24] and the US psoriasis study has been described elsewhere.[21]

ImmunoChip data on 2,425 AD case subjects, 3,580 psoriasis case subjects, and 9,061 control subjects were obtained from previous studies,[11,12] including data on a subset of case and control individuals also analyzed by GWAS. Results of analysis of the four most prevalent *FLG* (RefSeq accession number NM_002016.1) loss-of-function mutations were obtained for a total of 2,865 case subjects and 5,540 control subjects as data generated for previous studies;[11,20] the *FLG* mutations in these analyses are as follows: p.Arg501* (c.1501C>T), p.Ser761Cysfs*36 (c.2282_2285del), p.Arg2447* (c.7339C>T), and p.Ser3247* (c.9740C>A) (R501X, 2282del4, R2447X, and S3247X, respectively).

The institutional review board in each contributing center approved these studies. All participants (or their parents or guardians) gave written informed consent.

Study Design

The study design is summarized in Figure 1.

Quality Control

Quality control and standard GWAS analysis of genotyped single-nucleotide variants (SNVs) was carried out with PLINK[30] and R. Samples with extensive missing data (rate >5%), excess of heterozygosity or homozygosity, and discrepant gender determined on the basis of average X-chromosomal heterozygosity compared to the gender recorded in the database were excluded. We then examined identity-by-state (IBS) sharing and estimated identity-by-decent (IBD) on a pruned SNV set between all pairs of individuals and deleted resulting duplicates or closely related samples with PI_HAT > 0.1875 (halfway between expected IBD for third- and second-degree relatives). Multidimensional scaling (MDS) of the pairwise IBS matrix was carried out to identify and delete outliers of unusual ancestry and to calculate genome-wide principal-component scores for each individual. We excluded 894 samples because of SNVs showing a missing rate of >5%, deviation of Hardy-Weinberg equilibrium $p_{HWE} < 10^{-8}$, or minor allele frequency (MAF) <5% (summarized in Table S2). After quality control, the resulting SNVs and samples were analyzed for association via logistic regression with age, sex, and principal-component scores as covariates. Results from each panel were investigated to determine whether established GWAS loci were identified for the respective trait of interest, and genomic control inflation factors were calculated.

Imputation of SNVs and Classical HLA Alleles

Any SNVs showing significant association were checked (e.g., by visual inspection of the intensity cluster plots and investigation of consistency of LD with surrounding markers) and those SNVs deemed unreliable were removed. The final data sets of high-quality SNVs were prephased with SHAPEIT[31] and subsequently used to perform imputation with IMPUTE2,[32] the 1000 Genomes reference panel (integrated variant set, release March 2012).[33] In the Irish AD collection (Table S1), case and control subjects were genotyped on different platforms, and therefore only the 131,692 SNVs in common between the platforms were used to inform imputation.

Postimputation SNVs with low imputation quality (info score < 0.4), call rate <95%, deviation from $p_{HWE} < 10^{-8}$, or MAF < 5%

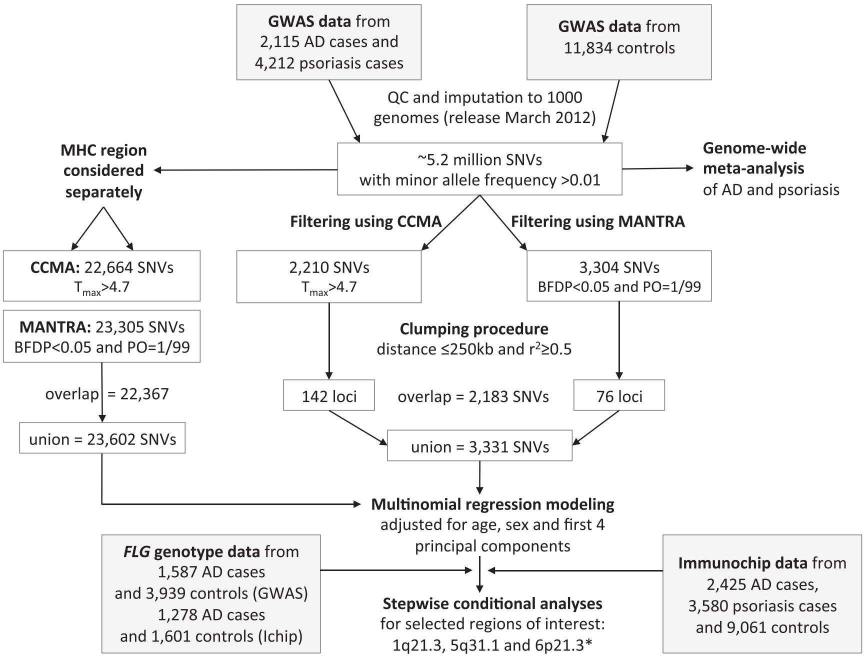

Figure 1. Study Design
Abbreviations are as follows: CCMA, case control meta-analysis; MANTRA, meta-analysis of trans-ethnic association studies; BFD, Bayesian false discovery; PO, prior odds; *conditional analysis for the MHC was also carried out with imputed classical HLA-allele (detailed in the Subjects and Methods).

were excluded. A final data set of approximately 5.2 million SNVs in 2,079 AD case subjects, 3,867 control subjects, 4,212 psoriasis case subjects, and 8,032 control subjects were eligible for subsequent analysis (Table S3).

Classical alleles for *HLA-A*, *HLA-B*, and *HLA-C* were imputed for each case-control cohort separately by HLA*IMP[34,35] and best guess genotypes with probability >0.9. Additional classical *HLA-DQA1*, *HLA-DQB1*, and *HLA-DRB1* alleles were imputed in each case-control cohort with the exception of the Irish samples, in which there were insufficient informative SNPs. Alleles with a frequency >1% were put forward for analysis. For each individual, alleles were coded as having no, one, or two copies of the respective allele via allele probability >0.9. We obtained high-quality data at the four-digit level with call rates of 92%–100% and accuracy of 92%–98%.

Statistical Analysis

Meta-GWAS was performed on each disease, via standard methodologies. To analyze these findings further, we developed two different meta-analysis-based approaches to filter SNVs and model the contrasting effects in each disease. The first was a compare and contrast meta-analysis (CCMA) approach inspired by a subset-based method.[36] The second used transethnic meta-analysis implemented in the MANTRA software,[37] combining all six studies by using prior clustering to reflect the ethnic difference and the disease type. The MHC region was reserved for separate analysis because of its unique and complex variability and patterns of strong linkage disequilibrium (LD).

The CCMA approach is based on an adaptation of an idea of Bhattacharjee et al.,[36] who modeled association with heterogeneous traits. With METAL,[38] we calculated z-scores signed positive or negative with respect to the same reference allele for two meta-analyses, T_1, combining AD studies only, and T_2, combining psoriasis studies only. We then calculated the overall test statistic T_{max} with the formula $T_{max} = \max(|T_1|, |T_2|, |T_{12shared}|, |T_{12opposing}|)$, where $T_{12shared} = (T_1 + T_2)/\sqrt{2}$ and $T_{12opposing} = (T_1 - T_2)/\sqrt{2}$. We categorized the effect of each SNV as corresponding to an effect on AD only, to an effect on psoriasis only, to a shared effect (in the same direction on AD and psoriasis), or to opposing effects, according to which of the four test statistics ($|T_1|, |T_2|, |T_{12shared}|, |T_{12opposing}|$) was the largest. In order to derive a p value for T_{max}, we worked out an empirical null distribution by simulating 10,000,000 realizations of two normally distributed random variables, Z_1 and Z_2. Then we calculated $Z_{12shared} = (Z_1 + Z_2)/\sqrt{2}$, $Z_{12opposing} = (Z_1 - Z_2)/\sqrt{2}$, and $Z_{max} = \max(|Z_1|, |Z_2|, |Z_{12shared}|, |Z_{12opposing}|)$. The emprical p values can be derived as $P_{emp} = (\#(Z_{max} > T_{max}) + 1)/(\#\ simulations + 1)$.

In a separate simulation of 1,000,000,000 replicates, we derived a calibration curve for the p values and found it suitable up to a p value of 10^{-9}. Hence with the calibration curve we can derive Z_{max} thresholds corresponding to standard genome-wide "suggestive" (10^{-5}) and genome-wide "significant" (10^{-8}) thresholds, corresponding to T_{max} values of approximately 4.7 and 6.0, respectively (Figure S1).

In the second approach we used the MANTRA software[37] developed for transethnic meta-analysis. MANTRA uses a Bayesian partition model for grouping studies according to their ethnicity. We adopted this idea and worked out a prior distribution to cluster studies according to both our phenotypes of interest and the genetic distance between the studies derived from our MDS analysis based on the pairwise IBS matrix: $D_{Total} = D_{Disease} + D_{Ethnicity}$, where $D_{Ethnicity}$ is a diagonal matrix of Euclidean distances between study centers. To distinguish the two diseases (psoriasis and AD), we set the corresponding cells of the $D_{Disease}$ matrix to $D_{ij} = 2 \times \max(D_{Ethnicity})$ and to account for the different subphenotype in AD (AD in general versus childhood AD), we set the corresponding cells of the $D_{Disease}$ matrix to $D_{ij} = \max(D_{Ethnicity})$, resulting in the prior components shown in Table S4.

We calibrated the resulting $\log_{10}\text{BF}_{\text{MANTRA}} = \log_{10}(\text{Bayes Factors})$ from the MANTRA software in order to find a threshold for filtering SNVs, which were compared with the CCMA top SNVs and subsequently carried forward to multinomial regression modeling. To perform this calibration, we calculated the Bayesian False Discovery Probability proposed by Wakefield[39] with diverse prior odds (PO) in favor of H_0:

$$\text{BFDP} = \frac{\text{BF}_{\text{MANTRA}} \times \text{PO}}{1 + \text{BF}_{\text{MANTRA}} \times \text{PO}}.$$

Sensitivity analysis was performed with only the $D_{Ethnicity}$ as prior matrix and we observe high correlation ($r^2 > 0.99$) of the top-ranked SNVs (BFDP < 0.05; PO $= 99$) with our analysis (data not shown).

Finally, we carried forward a filtered set of SNVs from CCMA and MANTRA for modeling via a multinomial regression model, adjusted for sex and the first four genome-wide principal-component scores. The multinomial model involves three outcome categories: the "baseline" category into which all controls are categorized, a "psoriasis" case category, and an "AD" case category (modeled through regression coefficients β_{PSO} and β_{AD}, respectively). This analysis makes use of individual-level genotypes and is thus more computationally intensive (although arguably more powerful and more statistically satisfactory) than CCMA and MANTRA. We calculated p values for tests that were designed to be sensitive to the following situations: an overall SNV effect (on either or both diseases, in either direction), an individual SNV effect on one disease (but not on the other), a shared SNV effect (operating in the same direction for both diseases), and a contrasting SNV effect (operating in opposing directions between both diseases), by performing Wald tests of the following linear hypotheses:

$$\text{Overall effect}: \quad H_0 : \begin{matrix} \beta_{PSO} + \beta_{AD} \\ \beta_{PSO} - \beta_{AD} \end{matrix} = \begin{matrix} 0 \\ 0 \end{matrix}, \quad H_1 : \begin{matrix} \beta_{PSO} + \beta_{AD} \\ \beta_{PSO} - \beta_{AD} \end{matrix} \neq \begin{matrix} 0 \\ 0 \end{matrix}$$

$$\text{Psoriasis effect}: \quad H_0 : \beta_{PSO} = 0, \quad H_1 : \beta_{PSO} \neq 0$$

$$\text{AD effect}: \quad H_0 : \beta_{AD} = 0, \quad H_1 : \beta_{AD} \neq 0$$

$$\text{Shared effect}: \quad H_0 : \beta_{PSO} + \beta_{AD} = 0, \quad H_1 : \beta_{PSO} + \beta_{AD} \neq 0$$

$$\text{Opposing effect}: \quad H_0 : \beta_{PSO} - \beta_{AD} = 0, \quad H_1 : \beta_{PSO} - \beta_{AD} \neq 0$$

The overall significance of the SNV was assessed through the 2 degree of freedom (df) test of overall effect, which compares the null hypothesis that the SNV has no effect on either psoriasis or AD with the alternative hypothesis that it has an effect on one or both diseases. The other four 1 df tests were used to categorize the effect (in analogy to CCMA) in four categories—AD only, psoriasis only, shared effect, and opposing effects—by categorizing according to the minimum of the p values: $p_{MNM} = \min(p_{AD}, p_{PSO}, p_{SHARED}, p_{OPPOSING})$. The rationale for the use of the minimum of these 1 df tests for categorization is as follows: if a SNV is associated with one disease but not the other, the test of a nonzero regression coefficient for that disease (even while unnecessarily also allowing for a nonzero coefficient for the other disease, as is done in the psoriasis effect and AD effect tests), should be more powerful than a test that erroneously groups together the coefficients of the associated and the nonassociated disease (as is done in the shared and opposing effect tests). This is on account of the fact that grouping together these coefficients will incur a penalty in terms of increasing the variance, while not incurring any greater expected magnitude of effect since the expected value of the regression coefficient for the nonassociated disease is zero. If, on the other hand, the SNV has effects that operate in the same direction on both diseases, then a test based on adding together these effects (as is done in the shared effect test) should be more powerful than considering each effect on its own, or subtracting one effect from the other (as is done in the opposing effect test), because adding together the coefficients induces the greatest magnitude of effect. Finally, if the SNV has effects that operate in opposite directions in the two diseases, then a test based on subtracting one effect from the other (as is done in the opposing effect test) should be most powerful because it induces the greatest magnitude of effect.

All analyses if not explicitly stated were carried out with R. For the purposes of this analysis, we distinguished between a shared genetic "region" and a shared genetic "locus." We arbitrarily designated a shared region as a block of genomic DNA spanning 2 Mb with association signals for both traits. We defined a genetic locus as the lead SNV and all SNVs with $r^2 > 0.5$.

Predicted Protein Network Analysis and Gene Ontology Analysis

Functional protein association networks were investigated in silico and gene ontology analyses were performed with $STRING_{9.1.}$

Results

Filtering Variants to Define Risk Effects

Quality control and imputation generated 5.2 million SNVs with a minor allele frequency >0.01 for further analysis (Figure 1). GWASs within each cohort resulted in genomic inflation factors λ between 1.03 and 1.08. Meta-GWAS performed on each disease confirmed previously reported risk loci in AD and psoriasis and illustrated areas of colocalization on chromosomes 1, 5, and 6 (Figure 2A).

Excluding the MHC, 2,210 SNVs were identified with shared (by which we mean alleles having effects operating in the same direction in both diseases), opposing, and disease-specific SNVs with CCMA test statistic $T_{max} > 4.7$. This threshold was defined to correspond to a suggestive significance of $p < 10^{-5}$ in order to reduce the probability of false negatives. The 2,210 SNVs were condensed to 142 distinct loci after an LD-based clumping procedure[30] with the following parameters: distance ≤ 250 kb and $r^2 \geq 0.5$.

Analysis with MANTRA revealed 3,304 SNVs with Bayesian false discovery probability (BFDP) < 0.05 with prior odds (PO) 1/99 resulting in 76 distinct loci after clumping. The overlap of CCMA and MANTRA gave 2,183 SNVs and the union of both methods resulted in 3,331 SNVs that were carried forward for multinomial regression modeling (MNM), which was adjusted for sex and the first four genome-wide principal-component scores. The results are displayed in Figure 2B, in which disease-specific, shared, and opposing loci are coded by color. SNVs showing genome-wide significance in at least one of the three methods of analysis ($T_{max} > 6$, BFDP < 0.05 with PO = 1/999, or $p_{MNM} < 10^{-8}$) map to 144 distinct loci (Table S5). Comparison of effect classification (AD, psoriasis, shared, opposing) in CCMA and MNM (Figure S2) showed an agreement of 94.8% when excluding the MHC region (Figure S3). For further investigation, we considered only loci containing more than one SNV and an effect classified in the same direction by CCMA and MNM.

Validation of Previously Reported AD- and Psoriasis-Risk Loci

15 European and 9 Asian loci have previously been reported in GWASs on AD, and 44 European and 9 Asian loci have been reported in association with psoriasis (Table S6). In our disease-specific meta-analysis individuals of

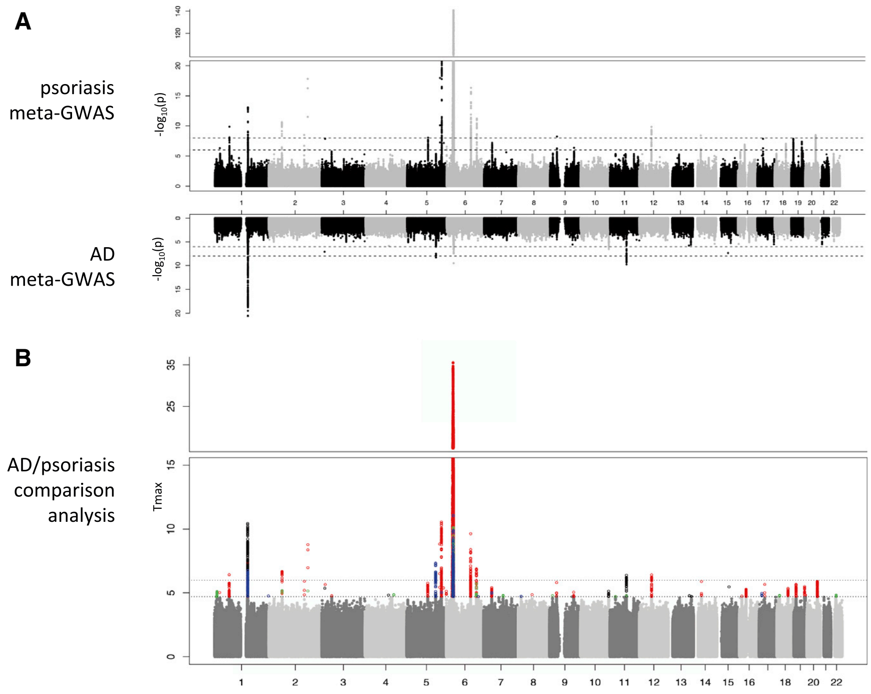

Figure 2. Genome-wide Comparison of AD and Psoriasis
(A) Mirrored Manhattan plots showing results of AD meta-GWAS (top) and psoriasis meta-GWAS (bottom).
(B) Comparative analysis of AD and psoriasis in which SNVs are color coded to show AD-specific effect (black), psoriasis-specific effect (red), shared effects defined as alleles operating in the same direction (green), and opposing effects (blue). The genome-wide significance level is marked at $p = 0.5 \times 10^{-8}$ ($T_{max} = 6.0$).

white European descent, 14 of the European AD loci as well as 43 of the psoriasis loci are replicated. Furthermore, 4 AD and 4 psoriasis loci so far reported only in Asians showed evidence for association in European populations ($p < 10^{-3}$): *CCDC80* (MIM 608298)/*CD200R1L* at 3q13.2, *CARD11* (MIM 607210) at 7p22.2, *ZNF365* (MIM 607818) at 10q21.2, and *BCAS1* (MIM 602968) at 20q13.2 in AD; *CSMD1* (MIM 608397) at 8p23.2, *SERPINB8* (MIM 601697) at 18q22.1, *MAMSTR* (MIM 610349)/*RASIP1* (MIM 609623) at 19q13.33, and *ZNF816A* at 19q13.41 in psoriasis (Table S6).

New Opposing-Effect Loci Identified by Genome-wide Comparative Analysis

Excluding the MHC, 25 loci showed a genome-wide significant association with either skin disorder, defined by all three methods of analysis (CCMA $T_{max} > 6$ and MANTRA BFDP < 0.05 with PO $= 1/999$, and $p_{MNM} < 10^{-8}$) including six loci that were coassociated with both AD and psoriasis. Each coassociated locus displayed opposing effects and two of these loci (2q31.2, 5q33.1) have not previously been reported as showing coassociation with AD and psoriasis (Table 1).

2q31.2 demonstrates an opposing effect at rs62176107 (MNM $p = 1.08 \times 10^{-34}$; Table S5); this variant is within exon 6 of *PRKRA* (MIM 603424) and also within microRNA 548n (MIR548N) and a noncoding transcript, AC009948.5. *PRKRA* encodes protein kinase interferon-inducible double-stranded RNA-dependent activator (PACT), a cellular dsRNA-binding protein originally identified as a binding partner and activator of PKR in response to extracellular stress.[46] More recently, it has been shown to be an essential factor in the PKR-independent initiation of RIG-I-induced antiviral response.[47] Of note, individuals with AD are known to be susceptible to viral skin infections, but cutaneous infections rarely occur in psoriasis.[48] MicroRNAs play a role in posttranscriptional regulation of gene expression by affecting the stability and translation of mRNAs, but the specific role of miRNA548n has not been defined. The most significantly associated ("lead") SNV at 2q31.2 (rs62176107, G>A, having the smallest p value from MNM) is a synonymous SNV with predicted effects on 12 transcripts, including *PRKRA* splice variants' UTR and intronic regions and a variant predicted to undergo nonsense-mediated decay (Ensembl release 75). Gene expression profiling data show downregulation of both *PRKRA* mRNA and miRNA548n in psoriatic lesions compared to nonlesional skin, but no significant differences in AD (Table S7).

The most highly significant variant at 5q33.1 (rs17728338) shows opposing effects on AD and psoriasis (MNM $p = 3.96 \times 10^{-38}$; Table S5) and lies 2 kb upstream of *ANXA6* (MIM 114070) and 8 kb downstream of *TNIP1* (MIM 607714). LD analysis in 1000 Genomes (release August 2009) via LocusZoom[49] showed that rs17728338 is located within a 25-kb block containing both *TNIP1* and *ANXA6*. The locus has previously been associated with psoriasis in European and Chinese populations but has not been implicated in AD. *TNIP1* is involved in TNF signaling and regulation of the transcription factor NF-κB;[21,50] it shows increased expression both in AD and psoriatic lesions compared to control skin (Table S7). In contrast, *ANXA6*, which encodes a calcium-dependent membrane and phospholipid binding protein, shows significant upregulation of expression in atopic skin compared to control skin (fold change 1.3, FDR $p = 0.016$) and lesional to nonlesional AD skin (fold change 2.4, $p = 0.027$), whereas expression is decreased in psoriatic versus healthy skin (fold change 0.7, $p = 6.38 \times 10^{-13}$) (Table S7). Clearly, further fine mapping is necessary to identify the causal variant that exerts opposing effects on AD and psoriasis, but we speculate that *ANXA6* might be a switch-point differentiating AD from psoriasis that

Table 1. Loci Showing Genome-wide Significant Association with Either AD or Psoriasis Defined by All Three Methods of Comparative Analysis

Chr Band	Reference SNV Number(s)	Position (hg19)	Nearest Gene(s) or Transcript(s)	Effect Observed in GW Analyses	Estimated Odds Ratio (95% CI)		Previous Report(s) of Association at This Locus
					AD	Psoriasis	
1p31.3	rs77614545 (del)	67749581	retro-DNAJB6 and *IL23R* (MIM 607562)	psoriasis	0.99 (0.92–1.07)	1.21 (1.15–1.28)	psoriasis: 1p31.3 locus, *IL28RA*[21,24,40]
1q21.3[a]	rs55879323	152168740	within *FLG-AS1*	opposing	0.76 (0.70–0.82)	1.05 (1.00–1.12)	AD and psoriasis: 1q21.3 locus, *HRNR*, *FLG*;[41] *FLG*;[20,42] *LCE3B*, *LCE3C*,[18] *LCE* gene cluster;[17] *LCE3D*[24]
	rs11205006, rs12144049	152440176, 152440910	RP1-91G5.3, *CRNN* (MIM 611312), *LCE5A* (MIM 612619)	AD	1.52 (1.41–1.64), 1.53 (1.42–1.64)	0.97 (0.92–1.03), 0.98 (0.92–1.03)	AD: 1q21.3 locus, *HRNR*, *FLG*;[41] *FLG*[20,42]
	rs471144	152454255	*LCE5A* (MIM 612619)	AD	1.54 (1.37–1.73)	1.03 (0.94–1.14)	AD: 1q21.3 locus, *HRNR*, *FLG*;[41] *FLG*[20,42]
	rs10888499	152532742	*LCE3E* (MIM 612617)	AD	1.49 (1.38–1.61)	0.98 (0.93–1.04)	AD: 1q21.3 locus, *HRNR*, *FLG*;[41] *FLG*[20,42]
	rs4112788	152551276	*LCE3D* (MIM 612616)	psoriasis	0.97 (0.90–1.05)	1.22 (1.15–1.28)	psoriasis: LCE gene cluster;[17] *LCE3D*[24]
	rs1581803	152592281	*LCE3A* (MIM 612613)	psoriasis	0.97 (0.90–1.04)	1.22 (1.15–1.30)	psoriasis: LCE gene cluster[17]
	rs77199844 (del)	152757094	*LCE1E* (MIM 612607)	[AD]	2.01 (1.72–2.35)	1.16 (1.01–1.33)	AD: 1q21.3 locus, *HRNR*, *FLG*;[41] *FLG*[20,42] psoriasis: LCE gene cluster[17]
	rs4363385	152989321	SNORA31, *SPRR3* (MIM 182271), *SPRR1B* (MIM 182266)	[opposing]	1.23 (1.15–1.32)	0.89 (0.85–0.94)	AD: *SPRR3* repeat number variant[43]
2p16.1	rs35741374	61072567	within lincRNA AC010733.4	psoriasis	1.09 (1.01–1.63)	1.20 (1.15–1.27)	psoriasis: *REL*;[24] *NR*[23]
2q31.2	rs62176107	179300971	exonic *PRKRA* and within miRNA548n and AC009948.5	opposing	0.55 (0.46–0.65)	1.42 (1.32–1.53)	–
5q31.1[a]	rs1295686	131952222	intronic *IL13* (MIM 147683) and within AC004041.2	[opposing]	1.27 (1.17–1.38)	0.88 (0.82–0.94)	AD and psoriasis: *IL13*;[21] *KIF3A*, *IL13*;[22] *KIF3A*, *IL4*, *IL13-RAD50*;[10] multiple effect locus *RAD50/IL13*;[20] *C5orf56*
	rs6596086	131995843	intronic *RAD50* (MIM 604040)	opposing	1.30 (1.20–1.41)	0.85 (0.8–0.91)	AD and psoriasis: *IL13-RAD50*;[10] multiple effect locus *RAD50/IL13*[20]
5q33.1	rs17728338	150478318	*ANXA6* (MIM 114070)	opposing	0.70 (0.59–0.84)	1.77 (1.61–1.95)	psoriasis: *TNIP1*[21,42]
5q33.3	rs10515778, rs7715173, rs7719425[b]	158658012, 158664631, 158670938	within CTB-11I22.1	psoriasis	1.07 (0.98–1.17)	1.29 (1.21–1.38)	psoriasis: 5q33.3 locus, *IL12B*[17,21,24,44]; AD: *PTTG1*[42]
	rs11135056, rs4921442[b]	158687281, 158694100	intronic *UBLCP1* (MIM 609867)	psoriasis	0.97 (0.89–1.05)	1.45 (1.35–1.56)	
	rs2546890	158759900	within AC008697.1	psoriasis	1.01 (0.94–1.06)	1.39 (1.32–1.47)	
	rs5872599 (indel)	158859989	lincRNA AC008703.1, *IL12B* (MIM 161561)	psoriasis	0.82 (0.73–0.93)	1.54 (1.45–1.64)	
6q21	rs9487605	111582885	intronic *KIAA1919*	psoriasis	1.06 (0.98–1.14)	1.27 (1.20–1.35)	–
	rs240993	111673714	intronic *REV3L* (MIM 602776)	psoriasis	1.05 (0.97–1.13)	1.29 (1.22–1.36)	–
	rs9481169	111929862	*TRAF3IP2* (MIM 607043)	psoriasis	0.98 (0.86–1.11)	1.58 (1.45–1.72)	psoriasis and psoriatic arthritis: *TRAF3IP2*[24,44,45]

(*Continued on next page*)

Table 1. *Continued*

Chr Band	Reference SNV Number(s)	Position (hg19)	Nearest Gene(s) or Transcript(s)	Effect Observed in GW Analyses	Estimated Odds Ratio (95% CI) AD	Psoriasis	Previous Report(s) of Association at This Locus
6q23.2	rs643177, rs582757[b]	138195693, 138197824	*TNFAIP3* (MIM 610669)	psoriasis	1.05 (0.97–1.14)	1.27 (1.20–1.34)	psoriasis: *TNFAIP3*[21,24]
11q13.5	rs2212434, rs7126418[b]	76281593, 76292573	*c11orf30* (MIM 608574)	AD	1.29 (1.21–1.39)	1.05 (1.00–1.11)	AD: *c11orf30-LRRC32*;[10,41] *c11orf30*;[22] 11q13 locus[20]
12q13.3	rs36207871 (del)	56684496	intronic *CS* (MIM 118950)	psoriasis	0.94 (0.83–1.06)	1.47 (1.33–1.67)	psoriasis: 12q13.3 locus, *IL23A*, *STAT2*;[21] *IL23A*[24]
	rs11575234	56744276	intronic *STAT2* (MIM 600556)	psoriasis	0.90 (0.79–1.02)	1.47 (1.32–1.64)	psoriasis: *STAT2*[21]

Genome-wide significance is defined as CCMA $T_{max} > 6$ and MANTRA BFDP < 0.05 with PO = 1/999 and multinomial model $p < 10^{-8}$; genes and transcripts identified from UCSC Genome Browser Human Feb. 2009 (GRCh37/hg19) Assembly accessed 21 March 2014; this variant of *FLG-AS1* extends across *HRHR* and *FLG*; RP1-91G5.3 extends across *CRNN*; AC004041.2 extends across *RAD50* and *IL13*; CTB-11I22.1 overlaps *RNF145*.
[a]1q21.3 and 5q31.1 were further investigated via stepwise conditional analysis (the results are shown in Table 2); square brackets indicate results from the univariate analysis that were subsequently accounted for by other nearby variants when examined by stepwise conditional analysis.
[b]Multiple SNVs are assigned to the same LD block but odds ratios and 95% CI are presented only for the first SNV.

reflects the importance of calcium-dependent effects in keratinocyte differentiation.

Opposing effect loci were also identified within regions characterized by complex patterns of LD within the EDC (Figure S4), the cytokine cluster on 5q31.1 (Figures S4 and S5), and the MHC. These regions were therefore investigated further via conditional analysis.

Stepwise Conditional Analysis within 1q21.3 and 5q31.1 Identifies Opposing and Disease-Specific Risk Variants

Coverage of the EDC was achieved via GWAS data (Figure 3), whereas ImmunoChip data provided better coverage for the cytokine cluster on 5q31.1 (Figures 4 and S5).

Within 1q21.3 we identified seven LD blocks with disease-specific or opposing signals (Figure 3A). Stepwise conditional analysis on the four most prevalent *FLG*-null mutations and variants tagging the *LCE3B-LCE3C* deletion identified one AD-specific locus mapping to *FLG*, a psoriasis-specific locus mapping to *LCE3B-LCE3C*, and a locus with opposing effects on both diseases mapping to *RPTN* (MIM 613259)/*HRNR*/*FLG-AS1* (Figure 3B and Table 2). After conditioning on the four *FLG*-null mutations and the *LCE3B-LCE3C* deletion, the G allele of the lead MNM SNV rs12130219 decreases the risk for AD ($OR_{ADcond} = 0.812$, $p_{ADcond} = 0.0018$) and increases the risk for psoriasis ($OR_{PSOcond} = 1.119$, $p_{PSOcond} = 3.68 \times 10^{-4}$) (Table 2). Filaggrin, repetin, and hornerin are all members of the S100 fused-type protein family and each contribute to the cornified cell envelope, a functional component of the epidermal barrier. Both *FLG* and *HRNR* show reduced expression in AD[51–53] whereas *RPTN* shows no significant difference (Table S7). In psoriasis *HRNR* expression can be downregulated[53] or upregulated,[54] *RPTN* expression might be upregulated, and *FLG* expression might be downregulated[55] or dysregulated[15] (Table S7). The function of *FLG-AS1* (*FLG* antisense RNA1) is currently undefined, but its proximity to *FLG* and *HRNR* suggests a role in coordinating keratinocyte terminal differentiation. *FLG-AS1* expression is increased in psoriasis lesional compared with nonlesional skin, whereas in AD lesional skin, expression is reduced (Table S7). Together, our results confirm the role of the *LCE3B-LCE3C* deletion in psoriasis and support the presence of genetic risk mechanisms for AD within the EDC in addition to the predominant effect of *FLG*-null mutations, with opposing effects on psoriasis.

Conditional analysis at 5q31.1 revealed three independent loci specifically contributing to AD risk: *IL13* (MIM 147683, rs848, $OR_{ADfull} = 1.12$, $p = 0.0204$), *KIF3A* (MIM 604683, rs 2299009, $OR_{ADfull} = 1.16$, $p = 4.1 \times 10^{-4}$), and *SLC22A4* (MIM 604190)/*C5orf56* (rs74458173, $OR_{ADfull} = 1.57$, $p = 2.0 \times 10^{-4}$) (Figure 4A, Table 2). None of these loci showed significant effects on psoriasis. However, a fourth independent locus has opposing effects on AD and psoriasis. The most highly significant variant maps

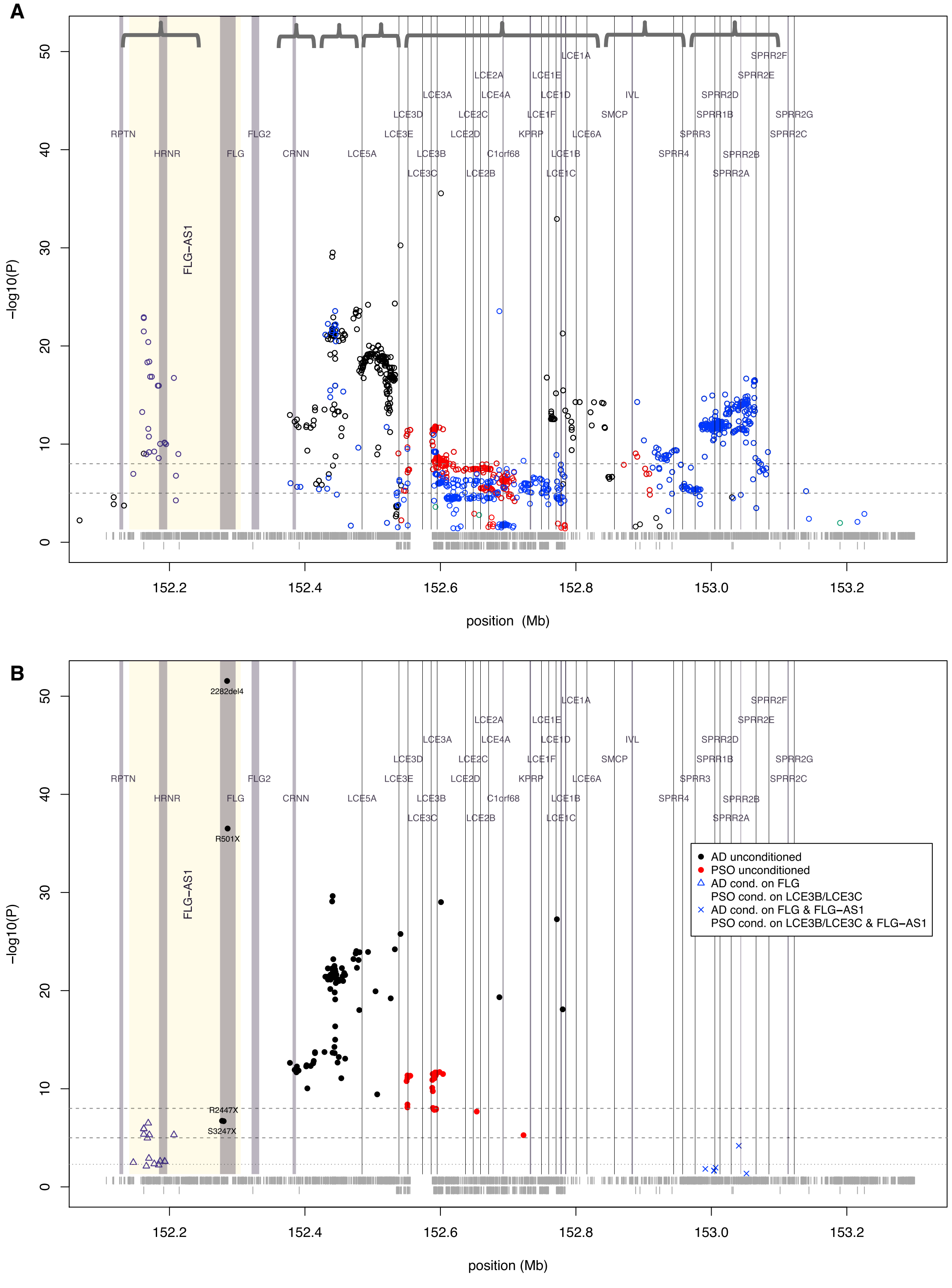

Figure 3. Regional Association within the Epidermal Differentiation Complex at 1q21.3

(A) Multinomial regression model with GWAS and ImmunoChip data. Seven blocks of linkage disequilibrium are indicated by curly brackets; black circles indicate AD-specific association, red circles indicate a psoriasis-specific association, blue circles represent opposing effects in AD and psoriasis, and green circles indicate shared effects. Vertical lines have been drawn to mark the positions of known genes and transcripts (identified from UCSC Genome Browser, GRCh37/hg19 accessed Feb. 2009) and the horizontal dotted lines indicate thresholds of suggestive and genome-wide significance ($p = 10^{-5}$ and 10^{-8}). The horizontal gray bands at the bottom indicate the coverage of the region by GWAS SNVs (upper row) and ImmunoChip SNVs (lower row).

(B) Conditional regional association plot of stepwise logistic regression using GWAS and ImmunoChip data. The different colored symbols indicate association results after each step of analysis, as follows. Unconditioned results are shown by black dots to indicate association with AD and red dots to indicate association with psoriasis; blue triangles and blue crosses represent results after conditioning on the known disease-associated variants, *FLG* in AD and *LCE3B-LCE3C* deletion in psoriasis; SNVs indicated by the same symbol are in LD

(legend continued on next page)

to *RAD50* (MIM 604040, rs6596086, $OR_{ADfull} = 1.17$, $OR_{PSOfull} = 0.88$, $p = 6.3 \times 10^{-7}$); this variant is associated with increased risk of AD but is protective against psoriasis (Figure 4B, Table 2).

Analysis of the MHC Confirms Multiple Psoriasis-Risk Loci and Identifies Opposing Effects

In the extended HLA region, we took forward 23,479 SNVs with $T_{max} > 4.7$ or BFDP < 0.05 (PO $= 1/99$) and 75 variables representing the classical HLA alleles obtained from HLA imputation for multinomial modeling, of which 18,515 SNVs were classified as specific to psoriasis by CCMA and MNM. To reduce the data set for post hoc analysis, we considered only SNVs with effect classification in the same direction by CCMA and MNM, meeting the $p < 10^{-5}$ threshold in MNM. Within the psoriasis-specific markers, we excluded all tagging SNVs ($r^2 > 0.8$ with the lead SNV), resulting in 1,503 SNVs, including those previously reported for AD.[20]

The strongest and most significant association was observed for psoriasis, a variant (rs111576655 $OR_{PSOfull} = 3.32$, $p = 3.2 \times 10^{-65}$) tagging the well-known psoriasis-risk allele HLA-C*06:02 ($OR_{PSOfull} = 3.59$, $p = 8.7 \times 10^{-154}$). Conditional analysis revealed two additional independent loci contributing to psoriasis risk at *MICA* (MIM 600169, rs201374403, $OR_{PSOfull} = 1.65$, $p = 1.0 \times 10^{-26}$) and *HLA-A* (MIM 142800, rs113573479, $OR_{PSOfull} = 1.41$, $p = 2.7 \times 10^{-17}$), as well as two loci with opposing effects at *HLA-C* (MIM 142840, rs1793889, $OR_{ADfull} = 0.6$, $OR_{PSOfull} = 1.18$, $p = 1.1 \times 10^{-9}$) and *HLA-DRB1* (MIM 142857, rs28383201, $OR_{ADfull} = 0.61$, $OR_{PSOfull} = 1.18$, $p = 6.5 \times 10^{-9}$) (Figure 5, Table 3). Conditional analysis with imputed classical alleles identified five independent HLA-class I alleles contributing to psoriasis risk in addition to HLA-C*06:02 and two alleles with opposing effects: HLA-C*03:03 ($OR_{ADfull} = 0.71$, $OR_{PSOfull} = 1.27$, $p = 2.3 \times 10^{-5}$) and HLA-DQA1*02:01 ($OR_{ADfull} = 0.64$, $OR_{PSOfull} = 1.09$, $p = 6.0 \times 10^{-8}$; $r^2 = 0.405$ with rs28383201) (Table 3).

Ontology and Network Analysis of Genes Indicate Effects in the Skin Barrier and Immune Response

Genes implicated from genome-wide and conditional analyses (identified from Tables 1, 2, and 3) were investigated via predicted protein network and gene ontology (GO) analysis. The results are summarized in Figure S6. The GO term "keratinocyte differentiation" (GO:0030216) is enriched in genes implicated in AD and psoriasis risk (FDR $p = 4.3 \times 10^{-4}$ in AD; $p = 6.9 \times 10^{-4}$ in psoriasis; and $p = 2.7 \times 10^{-3}$ in opposing effects). The GO term "response to interferon-gamma" (GO:0034341) is also significantly enriched in psoriasis (FDR $p = 1.9 \times 10^{-3}$).

Discussion

This genome-wide comparative analysis confirms a high degree of genomic coincidence between AD and psoriasis, suggesting that common molecular mechanisms are involved. This agrees with the central role of epidermal barrier defects and T-cell-dominated inflammation in both diseases.[48] Within the six regions of colocalization, we demonstrate coassociated and independent disease-specific loci. Of note, all coassociated loci display opposing (antagonistic) effects on AD and psoriasis, in agreement with the epidemiological observations of lower-than-expected coincidence between these diseases in the population.[5] Within these loci, specific variants including chromosome 2q31.2 (rs62176107), chromosome 5q33.1 (rs17728338), and within *RAD50* on chromosome 5q33.3 (rs6596086) demonstrate opposing effects on risk of AD and psoriasis. This raises the intriguing possibility that the same biological mechanisms might act differentially on AD versus psoriasis. However, our current data cannot distinguish this specific opposing mechanism from the possibility that each lead variant is in LD with other variants having opposing effects in each disease.

The majority of the opposing effect loci are implicated in pathways related to adaptive immunological functions, which potentially mirrors the polarized immune mechanisms.[6] It might further be speculated whether the presence of multiple opposing alleles reflects balancing selection as a response to heterogeneity in environmental pressures. Balancing selection is particularly common within the extended MHC region and has been proposed as a potential explanation for antagonistic effects at multiple loci in different autoimmune diseases.[56]

Two of the loci displaying opposing effects (*ANXA6/TNIP1* and *PRKRA*) have not previously been reported in association with psoriasis and/or AD. Formal external validation is limited by the requirement for additional independent, population-matched GWAS data for AD and psoriasis, but data available from RNA sequencing and microarray analyses provide some support for the differential expression of *ANXA6/TNIP1* and *PRKRA* in AD and psoriasis, relative to normal or uninvolved skin. The lead variant within *PRKRA* might mediate opposing effects in AD and psoriasis via miRNA processing and/or cellular response to environmental stress, and we hypothesize that this reflects the striking differential susceptibility to viral and bacterial skin infections observed in AD and psoriasis. The opposing effect of variation in *ANXA6* suggests a role for calcium-dependent effects in defining patterns of skin inflammation.

On chromosome 1q21.3, apart from well-established AD-associated *FLG* mutations and psoriasis-associated

with the lead SNV of each stepwise conditional analysis (defined as $r^2 \geq 0.5$). Vertical lines are drawn to mark the positions of known genes and transcripts (identified from the UCSC Genome Browser GRCh37/hg19 accessed Feb. 2009), and horizontal dotted lines indicate significance thresholds of $p = 0.005$, 10^{-5}, and 10^{-8}. The horizontal gray bands at the bottom indicate the coverage of the region by GWAS SNVs (upper row) and ImmunoChip SNVs (lower row).

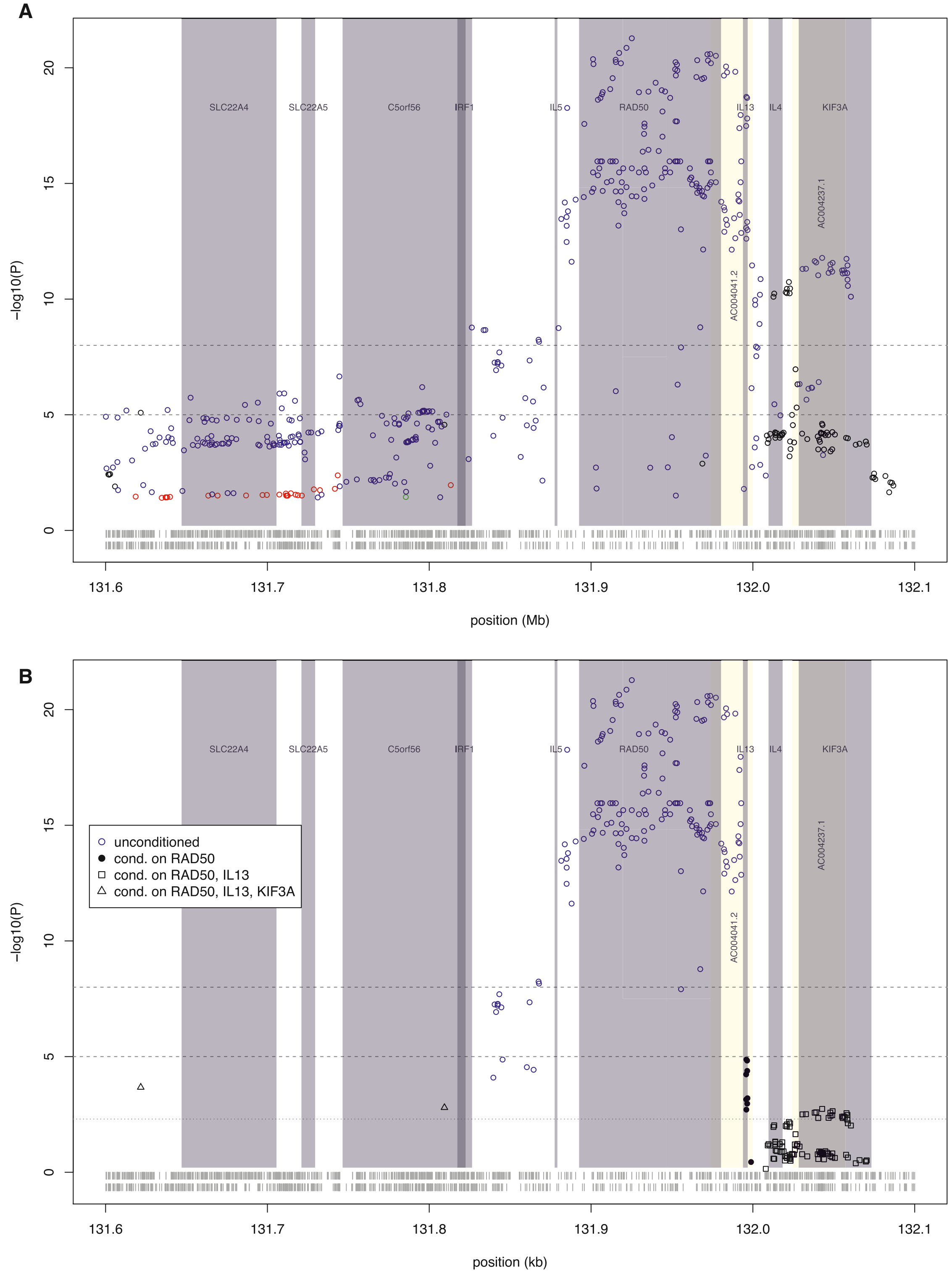

Figure 4. Regional Association within the Cytokine Cluster at 5q31.1

(A) Multinomial regression model with GWAS and ImmunoChip data. Black circles indicate AD-specific association, red circles indicate psoriasis-specific association, blue circles represent opposing effects in AD and psoriasis, and green circles indicate shared effects. Vertical gray shading marks the positions of known genes (identified from the UCSC Genome Browser GRCh37/hg19 accessed Feb. 2009), and horizontal dotted lines indicate suggestive and genome-wide significance thresholds ($p = 10^{-5}$ and 10^{-8}, respectively); results are shown for SNVs in LD with the lead SNV (defined as $r^2 \geq 0.5$). The horizontal bands at the bottom indicate the coverage of the region by GWAS SNVs (upper row) and ImmunoChip SNVs (lower row).

(B) Conditional regional association plot of the EDC by multinomial regression of GWAS and ImmunoChip data. Different symbols indicate association results after each step of analysis, as follows. Unconditioned results are shown by blue circles representing opposing effects in AD and psoriasis; black dots show AD-specific association results after conditioning on the lead SNV in *RAD50* (a gene reported to

(legend continued on next page)

deletion of *LCE3B-LCE3C*, *FLG-AS1* is a plausible candidate to mediate differential AD/psoriasis risk via the network of regulatory elements coordinating gene expression.[57] Natural antisense transcripts contribute to gene regulation via a variety of transcriptional and posttranscriptional mechanisms[58] and include effects on human epidermal differentiation.[59] The proximity of *FLG-AS1* to *FLG* and *HRNR*, combined with data showing coordinated differential expression of these genes, supports a role in control of keratinocyte terminal differentiation.

On chromosome 5q31.1, antagonistic signals for AD and psoriasis have previously been attributed to *IL13*.[10,11,21] We here show that *IL13* polymorphisms specifically influence AD risk, whereas opposing signals map to *RAD50*. The Rad50 protein, a component of the MRN complex (Mre11, Rad50, and Nbs1), is involved in DNA double-strand break repair but has no known function directly related to AD or psoriasis. However, *RAD50* mRNA shows significantly increased expression in psoriasis lesional skin and a trend to reduced expression in AD lesional skin (Table S7). Of note, *RAD50* is located in the center of the Th2-cytokine cluster and its 3′ end is part of a locus control region regulating expression of these cytokine genes.[60] AD and psoriasis represent opposing extremes of Th2 cell dysregulation, and therefore we hypothesize that *RAD50* polymorphisms might exert opposing effects on AD and psoriasis through variation in DNA repair resulting in a differential skew in Th2 cell response.

Our dissection of the MHC locus confirms the presence of multiple independent psoriasis-risk loci. Markers tagging HLA-Cw*0602 generate the strongest effects, which is in line with previous reports.[17,21,24,44,61] $CD8^+$ T cells are increased in the epidermis of lesional psoriatic skin, and the association of psoriasis susceptibility primarily with class I HLA alleles might reflect the critical role of psoriasis-associated (auto-)antigen presentation to pathogenic $CD8^+$ T cells.[62] $CD8^+$ T cells are also increased in the epidermis of AD skin, but with strikingly different cytokine profiles compared to psoriasis.[63] The opposing effects of class II HLA alleles in AD and psoriasis might represent the differential responses to pathogenic and allergenic peptides presented to $CD4^+$ T cells.[64] GWASs in AD by univariate and multivariate models have reported association signals in the MHC class I and II regions[22,65] and two specific HLA class II haplotypes, HLA-DRB1*0701 (a protective effect) and HLA-B*4402 (a risk effect).[20] Our analysis confirms the association of classical HLA class II alleles with AD, but in the conditional analysis, only HLA-DQA1*02:01 remained, showing a significant protective effect on AD and a significant opposing effect on psoriasis. A further opposing locus mapped to HLA-C*03:03 (Table 3).

The reported observation of AD occurring within the offspring of parents with psoriasis[8] is not supported by our findings, and the observation that both Th1-cell-dominated autoimmune and Th2-cell-dominated allergic diseases can show aggregation within families[7] also presents a discrepancy with our analyses. It is possible that there are shared risk loci for AD and psoriasis that were not detected in our current study because of lack of power, if the shared effect is not strong; alternatively, there might be hereditary risk factors associated with predisposition to any chronic inflammatory (auto-)immune disease. It is also possible that diagnostic misclassification occurs, particularly in pediatric cases, where the clinical signs of psoriasis are more difficult to distinguish from AD than is the case in adult disease,[66] or by recall bias for disease in parents.

It is interesting to estimate the extent to which our findings can explain the mutual exclusivity of AD and psoriasis, but an accurate assessment is hindered by the lack of published data on the proportion of AD and psoriasis cases where the diseases do and do not co-occur. Henseler et al. report a 25-fold lower prevalence of AD occurring in psoriasis cases[5] and assuming a prevalence of 10% and 2% for AD and psoriasis, respectively,[3,4] we estimate that the effects at the six opposing loci listed in Tables 1, 2, and 3 would result in a reduction in prevalence of AD from 10% to 8% within the group of individuals with psoriasis. This 2% reduction contrasts with the 25-fold reduction reported by Henseler et al.,[5] which is equivalent to a reduction of 9.6%, from 10% in the population to 0.4%. Our results have therefore explained approximately 21% (2/9.6 × 100) of the mutual exclusivity of AD and psoriasis.

Taken together, our comparative analyses of AD and psoriasis support a paradigm in which genetic factors determining keratinocyte differentiation and cutaneous barrier function have particularly strong effects on AD risk, whereas in psoriasis genetic factors influencing (auto-)antigen recognition are of paramount importance. Furthermore, multiple pleiotropic loci with antagonistic effects contribute to opposing mechanisms of adaptive immunity in both AD and psoriasis.

The meta-analysis-inspired methodology developed in the course of this study has demonstrated the power to leverage additional information from GWAS and high-density SNV data and to dissect cross-phenotype associations. AD and psoriasis are particularly well suited to the compare/contrast approach, but this methodology will

be associated with AD and psoriasis); black squares indicate the residual AD-specific association after conditioning on the lead SNVs in *RAD50* and *IL13* (genes reported to be associated with AD); and black triangles indicate the residual AD-specific association after additionally conditioning on the lead SNV in *KIF3A* (a gene reported to be associated with AD). SNVs indicated by the same symbol are in LD with the lead SNV of each stepwise conditional analysis (defined as $r^2 \geq 0.5$). Vertical gray shading marks the positions of known genes (identified from the UCSC Genome Browser GRCh37/hg19 accessed Feb. 2009), and horizontal dotted lines indicate significance thresholds of $p = 0.005$, 10^{-5}, and 10^{-8}; results are shown for SNVs in LD with the lead SNV (defined as $r^2 \geq 0.5$). The horizontal bands at the bottom indicate the coverage of the region by GWAS SNVs (upper row) and ImmunoChip SNVs (lower row).

Table 2. Conditional Analysis of the 1q21.3 and 5q31.1 Regions Showing Disease-Specific and Opposing Risk Effects in AD and Psoriasis

Data Source	Effect	SNV	Pos (hg19)	Allele	Candidate Genes	AD OR (95% CI)	AD p	Psoriasis OR (95% CI)	Psoriasis p	$p_{overall}$[a]	AD OR (95% CI)	AD p	Psoriasis OR (95% CI)	Psoriasis p	$p_{overall}$[a]
**Chromosome 1q21.3**															
						**Unconditioned Analysis**					**Conditional Analysis**[b]				
GWAS	Opposing	rs12130219	152162106	G/A	*FLG-AS1/ RPTN/HRNR*	0.66 (0.60–0.73)	1.1×10^{-16}	1.15 (1.09–1.224)	4.0×10^{-6}	1.2×10^{-23}	0.812 (0.71–0.93)	0.0018	1.119 (1.05–1.19)	3.68×10^{-4}	2.4×10^{-6}
GWAS	AD	rs12144049	152440910	C/T	*FLG*	1.53 (1.42–1.64)	2.7×10^{-30}	0.98 (0.92–1.03)	0.4140	3.0×10^{-30}	–	–	–	–	
GWAS	Psoriasis	rs1581803[c]	152592281	G/T	*LCE3B/ LCE3D*	0.97 (0.90–1.04)	0.4396	1.22 (1.16–1.30)	1.5×10^{-12}	1.6×10^{-12}	–	–	–	–	
GWAS	Opposing	rs35722864	153040505	G/GA	*SPRR* cluster	0.81 (0.75–0.88)	1.0×10^{-7}	1.129 (1.07–1.20)	2.1×10^{-5}	4.8×10^{-13}	0.851 (0.71–0.93)	0.0019	1.074 (1.01–1.14)	0.0211	1.3×10^{-4}
**Chromosome 5q31.1**															
						**Conditional Models**[d]					**Full Model**				
Ichip	Opposing	rs6596086	131952222	C/T	*RAD50*	1.31 (1.22–1.41)	1.7×10^{-13}	0.86 (0.80–0.92)	1.7×10^{-5}	5.7×10^{-21}	1.17 (1.07–1.28)	4.04×10^{-4}	0.88 (0.81–0.96)	0.0023	6.3×10^{-7}
Ichip	AD	rs848	131996500	A/C	*IL13*	1.20 (1.10–1.30)	5.6×10^{-5}	0.96 (0.89–1.04)	0.3375	4.14×10^{-5}	1.12 (1.02–1.23)	0.0197	0.96 (0.88–1.05)	0.3515	0.0204
Ichip	AD	rs2299009	132042813	G/T	*IL4/KIF3A*	1.14 (1.06–1.23)	7.9×10^{-4}	0.99 (0.92–1.06)	0.7392	0.0018	1.16 (1.07–1.25)	2.03×10^{-4}	0.99 (0.92–1.06)	0.6657	4.1×10^{-4}
Ichip	AD	rs74458173	131621731	A/C	*SLC22A4*	1.57 (1.26–1.96)	6.1×10^{-5}	1.02 (0.80–1.30)	0.8590	2.14×10^{-4}	1.57 (1.26–1.96)	5.71×10^{-5}	1.02 (0.80–1.30)	0.8683	2.0×10^{-4}

Full model incorporates the combined effects of independent SNVs identified by stepwise analyses.
[a]$p_{overall}$ represents the overall opposing signal calculated using the $T_{12opposing}$ statistic and derive the p value from the normal distribution.
[b]Conditional analysis of chr1q21.3 was conditioned on *FLG* for AD and *LCE3B/LCE3D* for psoriasis.
[c]rs1581803 tags the previously reported psoriasis SNV rs4112788 ($r^2 = 0.995$).
[d]Stepwise conditional analysis at chr5q31.1 was carried out using multinomial regression models and resulted in three additional signals for AD; this table shows only independent loci ($r^2 < 0.5$) and the SNV with the strongest association; the effect allele is underlined.

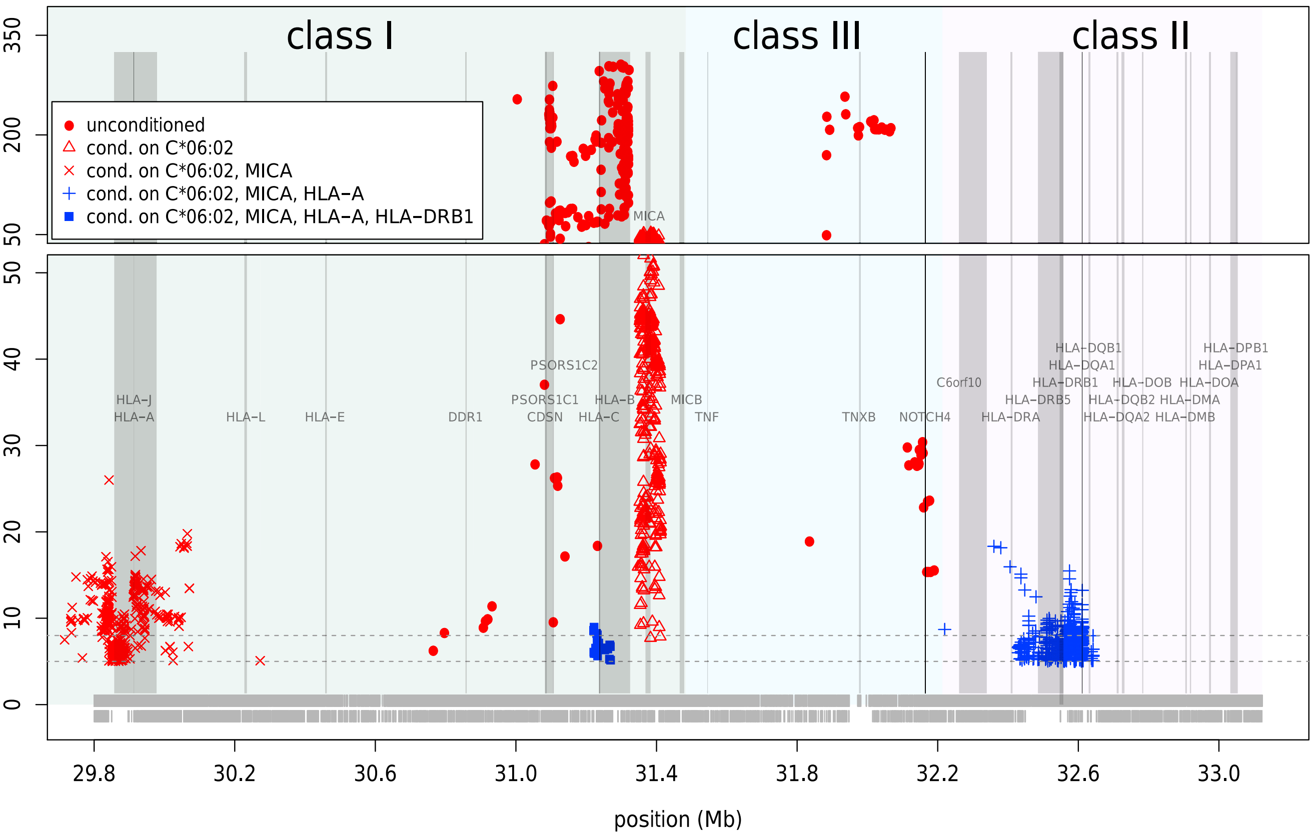

Figure 5. Conditional Regional Association within the Major Histocompatibility Complex at 6p21–22 via GWAS and ImmunoChip Data

Symbols indicate association results after each step of analysis, as follows. Unconditioned psoriasis-specific results are shown by red dots; red triangles show psoriasis-specific association results after conditioning on C*06:02 (known to be strongly associated with psoriasis); red ×s indicate psoriasis-specific association after conditioning on C*06:02 and *MICA*; blue +s indicate the association after conditioning on C*06:02, *MICA*, and *HLA-A* with opposing effects on AD and psoriasis; and blue squares indicate the residual association after conditioning on C*06:02, *MICA*, *HLA-A*, and *HLA-DRB1* with opposing effects on AD and psoriasis. SNVs indicated by the same symbol are in LD with the lead SNV of each stepwise conditional analysis (defined as $r^2 \geq 0.5$). Vertical shading marks the positions of known genes (identified from the UCSC Genome Browser GRCh37/hg19 accessed Feb. 2009) and HLA classes; horizontal dotted lines indicate significance thresholds of $p = 10^{-5}$ and $p = 10^{-8}$; results are shown for SNVs in LD with the lead SNV (defined as $r^2 \geq 0.5$). The horizontal bands at the bottom indicate the coverage of the region by GWAS SNVs (upper row) and ImmunoChip SNVs (lower row).

be applicable to many other complex traits with overlapping and disease-specific phenotypic features. Characterizing shared and opposing molecular mechanisms across complex phenotypes will expand our understanding of biology and disease and will have implications for treatment and drug discovery.

Supplemental Data

Supplemental Data include Supplemental Consortia Information, six figures, and seven tables and can be found with this article online at http://dx.doi.org/10.1016/j.ajhg.2014.12.004.

Consortia

Membership of the PAGE (Population Architecture using Genomics and Epidemiology) consortium is as follows: Trilokraj Tejasvi, Johann E. Gudjonsson, John J. Voorhees, Jun Ding, Yanming Li, Hyun M. Kang, Goncalo R. Abecasis, Dafna D. Gladman, Fawnda J. Pellett, Vinod Chandran, Cheryl F. Rosen, Proton Rahman, Sulev Koks, Külli Kingo, Tonu Esko, Andres Metspalu, Peter Gregersen, Andrew Henschel, Marin Aurand, Bruce Bebo, and Henry W. Lim.

Acknowledgments

We are grateful to the individuals who provided clinical data and DNA for research. This study makes use of genome-wide analysis data generated by the Wellcome Trust Case-Control Consortium (WTCCC); a full list of investigators is available at http://www.wtccc.org.uk. Funding for the WTCCC was provided by Wellcome Trust awards 076113 and 085475. This work was additionally supported by the Wellcome Trust through an Intermediate Clinical Fellowship (WT086398MA) to S.J.B., Senior Research Fellowship in Basic Biomedical Science (087436/Z/08/Z and 102858/Z/13/Z) to H.J.C., Programme Grant (092530/Z/10/Z) to W.H.I.M. and A.D.I., and Strategic Award (098439/Z/12/Z) to W.H.I.M. The KORA research platform was initiated and financed by the Helmholtz Zentrum München - German Research Center for Environmental Health, funded by the German Federal Ministry of Education and Research and by the State of Bavaria. KORA research was supported within the Munich Center of Health Sciences (MC

Table 3. Conditional Analysis of the MHC Region on 6p21–22 Showing Psoriasis-Specific and Opposing Risk Effects in AD and Psoriasis

						Conditional Models[a]					Full Model				
						AD		Psoriasis			AD		Psoriasis		
Data Source	Effect	SNV	Pos (hg19)	Allele	HLA Allele/ Candidate Genes	OR (95% CI)	p	OR (95% CI)	p	$p_{overall}$	OR (95% CI)	p	OR (95% CI)	p	$p_{overall}$
GWAS	PSO	rs111576655	31242731	A/T	C*06:02	0.84 (0.74–0.95)	0.0053	4.41 (4.10–4.74)	3.1×10^{-376}	9.8×10^{-380}	1.12 (0.81–1.54)	0.5071	3.32 (2.90–3.81)	2.3×10^{-69}	3.2×10^{-65}
GWAS	PSO	rs201374403	31383754	T/TAG	MICA	0.78 (0.7–0.88)	6.4×10^{-5}	1.68 (1.56–1.8)	7.9×10^{-48}	2.2×10^{-53}	0.81 (0.67–0.96)	0.0174	1.65 (1.50–1.81)	1.8×10^{-25}	1.0×10^{-26}
GWAS	PSO	rs113573479	29842444	A/G	HLA-A	0.89 (0.81–0.97)	0.0109	1.39 (1.30–1.49)	6.6×10^{-25}	1.0×10^{-26}	0.92 (0.81–1.04)	0.1948	1.41 (1.30–1.52)	2.8×10^{-17}	2.7×10^{-17}
GWAS	opposing	rs28383201	32574869	C/G	HLA-DRB1	0.59 (0.51–0.68)	4.6×10^{-13}	1.15 (1.06–1.24)	4.5×10^{-4}	3.3×10^{-16}	0.61 (0.52–0.71)	3.4×10^{-10}	1.18 (1.08–1.28)	1.0×10^{-4}	6.5×10^{-14}
GWAS	opposing	rs1793889	31222181	A/G	HLA-C	0.60 (0.50–0.73)	2.5×10^{-7}	1.18 (1.07–1.31)	0.0011	1.1×10^{-9}	0.60 (0.50–0.73)	2.5×10^{-7}	1.18 (1.07–1.31)	0.0011	1.1×10^{-9}

				Conditional Models[a]					Full Model				
				AD		Psoriasis			AD		Psoriasis		
Data Source	Effect	HLA Allele	HLA Allele Frequency in Ps/AD/Controls	OR (95% CI)	p	OR (95% CI)	p	$p_{overall}$	OR (95% CI)	p	OR (95% CI)	p	$p_{overall}$
GWAS	PSO	C*06:02	0.271/0.075/0.089	0.81 (0.71–0.91)	8.48×10^{-4}	4.28 (3.98–4.61)	2.9×10^{-362}	1.30×10^{-368}	0.97 (0.82–1.15)	0.7475	3.59 (3.26–3.95)	2.1×10^{-159}	8.7×10^{-154}
GWAS	PSO	A*02:01	0.28/0.227/0.239	0.95 (0.88–1.03)	0.1793	1.32 (1.24–1.40)	4.1×10^{-20}	1.1×10^{-20}	0.99 (0.90–1.08)	0.7739	1.32 (1.24–1.41)	1.8×10^{-17}	1.1×10^{-16}
GWAS	PSO	B*57:01	0.13/0.017/0.032	0.61 (0.46–0.80)	3.83×10^{-4}	1.63 (1.44–1.84)	8.0×10^{-15}	5.8×10^{-20}	0.60 (0.42–0.85)	0.0039	1.58 (1.38–1.81)	3.8×10^{-11}	1.1×10^{-13}
GWAS	PSO	C*12:03	0.048/0.044/0.038	0.87 (0.74–1.03)	0.1169	1.74 (1.54–2.00)	5.2×10^{-18}	3.5×10^{-19}	0.86 (0.72–1.04)	0.1295	1.85 (1.62–2.11)	7.5×10^{-20}	1.8×10^{-20}
GWAS	PSO	B*27:05	0.032/0.02/0.025	0.80 (0.63–1.01)	0.0619	1.59 (1.37–1.86)	2.9×10^{-9}	3.2×10^{-10}	0.74 (0.55–1.00)	0.0499	1.50 (1.28–1.76)	7.3×10^{-7}	1.8×10^{-7}
GWAS	PSO	A*01:01	0.221/0.154/0.166	1.03 (0.94–1.13)	0.5475	1.25 (1.17–1.35)	2.3×10^{-10}	1.7×10^{-9}	1.08 (0.96–1.21)	0.2013	1.23 (1.14–1.33)	6.8×10^{-8}	4.0×10^{-7}
GWAS	opposing	C*03:03	0.037/0.025/0.038	0.66 (0.54–0.81)	9.71×10^{-5}	1.30 (1.13–1.49)	2.5×10^{-4}	5.9×10^{-8}	0.71 (0.56–0.90)	0.0040	1.27 (1.10–1.47)	0.0011	2.3×10^{-5}
GWAS	opposing	DQA1*02:01	0.186/0.055/0.098	0.64 (0.54–0.76)	3.44×10^{-7}	1.09 (1.01–1.19)	0.0385	6.0×10^{-8}	0.64 (0.54–0.76)	3.4×10^{-7}	1.09 (1.01–1.19)	0.0385	6.0×10^{-8}

Effect allele is underlined. Abbreviations are as follows: PSO, psoriasis; AD, atopic dermatitis. Table shows only independent loci ($r^2 < 0.5$) and the SNV with the strongest association.
[a]Stepwise conditional analysis was carried out with multinomial regression models and resulted in three psoriasis-specific and two opposing signals.

Health), Ludwig-Maximilians-Universität, as part of LMUinnovativ. The project received infrastructure support through DFG Clusters of Excellence "Inflammation at Interfaces" (grants EXC306 and EXC306/2) and the German Federal Ministry of Education and Research within the framework of e:Med research and funding concept (sysINFLAME, 01ZX1306A). M.M.N. is a member of the Cluster of Excellence "ImmunoSensation." The NCRC-ADC is supported by the National Children's Research Centre, Dublin. We also acknowledge use of Trinity Biobank samples from the Irish Blood Transfusion Service. This work was supported by the NIH (R01AR042742, R01AR050511, R01AR054966, R01AR062886-01, R01AR062382) and by the Babcock Memorial Trust. J.T.E. is supported by the Ann Arbor Veterans Affairs Hospital.

Received: September 5, 2014
Accepted: December 5, 2014
Published: January 8, 2015

Web Resources

The URLs for data presented herein are as follows:

1000 Genomes, http://browser.1000genomes.org
Ensembl Genome Browser, http://www.ensembl.org/index.html
Online Mendelian Inheritance in Man (OMIM), http://www.omim.org/
PLINK, http://pngu.mgh.harvard.edu/~purcell/plink/
R statistical software, http://www.r-project.org/
RefSeq, http://www.ncbi.nlm.nih.gov/RefSeq
STRING 9.1, http://www.string-db.org/

References

1. Deckert, S., Kopkow, C., and Schmitt, J. (2014). Nonallergic comorbidities of atopic eczema: an overview of systematic reviews. Allergy *69*, 37–45.
2. Griffiths, C.E., and Barker, J.N. (2007). Pathogenesis and clinical features of psoriasis. Lancet *370*, 263–271.
3. Parisi, R., Symmons, D.P., Griffiths, C.E., and Ashcroft, D.M.; Identification and Management of Psoriasis and Associated ComorbidiTy (IMPACT) project team (2013). Global epidemiology of psoriasis: a systematic review of incidence and prevalence. J. Invest. Dermatol. *133*, 377–385.
4. Deckers, I.A., McLean, S., Linssen, S., Mommers, M., van Schayck, C.P., and Sheikh, A. (2012). Investigating international time trends in the incidence and prevalence of atopic eczema 1990-2010: a systematic review of epidemiological studies. PLoS ONE *7*, e39803.
5. Henseler, T., and Christophers, E. (1995). Disease concomitance in psoriasis. J. Am. Acad. Dermatol. *32*, 982–986.
6. Eyerich, S., Onken, A.T., Weidinger, S., Franke, A., Nasorri, F., Pennino, D., Grosber, M., Pfab, F., Schmidt-Weber, C.B., Mempel, M., et al. (2011). Mutual antagonism of T cells causing psoriasis and atopic eczema. N. Engl. J. Med. *365*, 231–238.
7. Hemminki, K., Li, X., Sundquist, K., and Sundquist, J. (2009). Shared familial aggregation of susceptibility to autoimmune diseases. Arthritis Rheum. *60*, 2845–2847.
8. Maas, T., Nieuwhof, C., Passos, V.L., Robertson, C., Boonen, A., Landewé, R.B., Voncken, J.W., Knottnerus, J.A., and Damoiseaux, J.G. (2014). Transgenerational occurrence of allergic disease and autoimmunity: general practice-based epidemiological research. Prim. Care Respir. J. *23*, 14–21.
9. Cookson, W.O., Ubhi, B., Lawrence, R., Abecasis, G.R., Walley, A.J., Cox, H.E., Coleman, R., Leaves, N.I., Trembath, R.C., Moffatt, M.F., and Harper, J.I. (2001). Genetic linkage of childhood atopic dermatitis to psoriasis susceptibility loci. Nat. Genet. *27*, 372–373.
10. Paternoster, L., Standl, M., Chen, C.M., Ramasamy, A., Bønnelykke, K., Duijts, L., Ferreira, M.A., Alves, A.C., Thyssen, J.P., Albrecht, E., et al.; Australian Asthma Genetics Consortium (AAGC); Genetics of Overweight Young Adults (GOYA) Consortium; EArly Genetics & Lifecourse Epidemiology (EAGLE) Consortium (2012). Meta-analysis of genome-wide association studies identifies three new risk loci for atopic dermatitis. Nat. Genet. *44*, 187–192.
11. Ellinghaus, D., Baurecht, H., Esparza-Gordillo, J., Rodríguez, E., Matanovic, A., Marenholz, I., Hübner, N., Schaarschmidt, H., Novak, N., Michel, S., et al. (2013). High-density genotyping study identifies four new susceptibility loci for atopic dermatitis. Nat. Genet. *45*, 808–812.
12. Tsoi, L.C., Spain, S.L., Knight, J., Ellinghaus, E., Stuart, P.E., Capon, F., Ding, J., Li, Y., Tejasvi, T., Gudjonsson, J.E., et al.; Collaborative Association Study of Psoriasis (CASP); Genetic Analysis of Psoriasis Consortium; Psoriasis Association Genetics Extension; Wellcome Trust Case Control Consortium 2 (2012). Identification of 15 new psoriasis susceptibility loci highlights the role of innate immunity. Nat. Genet. *44*, 1341–1348.
13. Palmer, C.N., Irvine, A.D., Terron-Kwiatkowski, A., Zhao, Y., Liao, H., Lee, S.P., Goudie, D.R., Sandilands, A., Campbell, L.E., Smith, F.J., et al. (2006). Common loss-of-function variants of the epidermal barrier protein filaggrin are a major predisposing factor for atopic dermatitis. Nat. Genet. *38*, 441–446.
14. Rodríguez, E., Baurecht, H., Herberich, E., Wagenpfeil, S., Brown, S.J., Cordell, H.J., Irvine, A.D., and Weidinger, S. (2009). Meta-analysis of filaggrin polymorphisms in eczema and asthma: robust risk factors in atopic disease. J. Allergy Clin. Immunol. *123*, 1361–1370, e7.
15. Hüffmeier, U., Traupe, H., Oji, V., Lascorz, J., Ständer, M., Lohmann, J., Wendler, J., Burkhardt, H., and Reis, A. (2007). Loss-of-function variants of the filaggrin gene are not major susceptibility factors for psoriasis vulgaris or psoriatic arthritis in German patients. J. Invest. Dermatol. *127*, 1367–1370.
16. Zhao, Y., Terron-Kwiatkowski, A., Liao, H., Lee, S.P., Allen, M.H., Hull, P.R., Campbell, L.E., Trembath, R.C., Capon, F., Griffiths, C.E., et al. (2007). Filaggrin null alleles are not associated with psoriasis. J. Invest. Dermatol. *127*, 1878–1882.
17. Zhang, X.J., Huang, W., Yang, S., Sun, L.D., Zhang, F.Y., Zhu, Q.X., Zhang, F.R., Zhang, C., Du, W.H., Pu, X.M., et al. (2009). Psoriasis genome-wide association study identifies susceptibility variants within LCE gene cluster at 1q21. Nat. Genet. *41*, 205–210.
18. de Cid, R., Riveira-Munoz, E., Zeeuwen, P.L., Robarge, J., Liao, W., Dannhauser, E.N., Giardina, E., Stuart, P.E., Nair, R., Helms, C., et al. (2009). Deletion of the late cornified envelope LCE3B and LCE3C genes as a susceptibility factor for psoriasis. Nat. Genet. *41*, 211–215.
19. Bergboer, J.G., Zeeuwen, P.L., Irvine, A.D., Weidinger, S., Giardina, E., Novelli, G., Den Heijer, M., Rodriguez, E., Illig, T., Riveira-Munoz, E., et al. (2010). Deletion of Late Cornified Envelope 3B and 3C genes is not associated with atopic dermatitis. J. Invest. Dermatol. *130*, 2057–2061.

20. Weidinger, S., Willis-Owen, S.A., Kamatani, Y., Baurecht, H., Morar, N., Liang, L., Edser, P., Street, T., Rodriguez, E., O'Regan, G.M., et al. (2013). A genome-wide association study of atopic dermatitis identifies loci with overlapping effects on asthma and psoriasis. Hum. Mol. Genet. *22*, 4841–4856.
21. Nair, R.P., Duffin, K.C., Helms, C., Ding, J., Stuart, P.E., Goldgar, D., Gudjonsson, J.E., Li, Y., Tejasvi, T., Feng, B.J., et al.; Collaborative Association Study of Psoriasis (2009). Genome-wide scan reveals association of psoriasis with IL-23 and NF-kappaB pathways. Nat. Genet. *41*, 199–204.
22. Hirota, T., Takahashi, A., Kubo, M., Tsunoda, T., Tomita, K., Sakashita, M., Yamada, T., Fujieda, S., Tanaka, S., Doi, S., et al. (2012). Genome-wide association study identifies eight new susceptibility loci for atopic dermatitis in the Japanese population. Nat. Genet. *44*, 1222–1226.
23. Stuart, P.E., Nair, R.P., Ellinghaus, E., Ding, J., Tejasvi, T., Gudjonsson, J.E., Li, Y., Weidinger, S., Eberlein, B., Gieger, C., et al. (2010). Genome-wide association analysis identifies three psoriasis susceptibility loci. Nat. Genet. *42*, 1000–1004.
24. Strange, A., Capon, F., Spencer, C.C., Knight, J., Weale, M.E., Allen, M.H., Barton, A., Band, G., Bellenguez, C., Bergboer, J.G., et al.; Genetic Analysis of Psoriasis Consortium & the Wellcome Trust Case Control Consortium 2 (2010). A genome-wide association study identifies new psoriasis susceptibility loci and an interaction between HLA-C and ERAP1. Nat. Genet. *42*, 985–990.
25. Williams, H.C., Burney, P.G., Pembroke, A.C., and Hay, R.J. (1994). The U.K. Working Party's Diagnostic Criteria for Atopic Dermatitis. III. Independent hospital validation. Br. J. Dermatol. *131*, 406–416.
26. Krawczak, M., Nikolaus, S., von Eberstein, H., Croucher, P.J., El Mokhtari, N.E., and Schreiber, S. (2006). PopGen: population-based recruitment of patients and controls for the analysis of complex genotype-phenotype relationships. Community Genet. *9*, 55–61.
27. Wichmann, H.E., Gieger, C., and Illig, T.; MONICA/KORA Study Group (2005). KORA-gen—resource for population genetics, controls and a broad spectrum of disease phenotypes. Gesundheitswesen *67* (1), S26–S30.
28. Weiland, S.K., von Mutius, E., Hirsch, T., Duhme, H., Fritzsch, C., Werner, B., Hüsing, A., Stender, M., Renz, H., Leupold, W., and Keil, U. (1999). Prevalence of respiratory and atopic disorders among children in the East and West of Germany five years after unification. Eur. Respir. J. *14*, 862–870.
29. O'Donovan, M.C., Craddock, N., Norton, N., Williams, H., Peirce, T., Moskvina, V., Nikolov, I., Hamshere, M., Carroll, L., Georgieva, L., et al.; Molecular Genetics of Schizophrenia Collaboration (2008). Identification of loci associated with schizophrenia by genome-wide association and follow-up. Nat. Genet. *40*, 1053–1055.
30. Purcell, S., Neale, B., Todd-Brown, K., Thomas, L., Ferreira, M.A., Bender, D., Maller, J., Sklar, P., de Bakker, P.I., Daly, M.J., and Sham, P.C. (2007). PLINK: a tool set for whole-genome association and population-based linkage analyses. Am. J. Hum. Genet. *81*, 559–575.
31. Delaneau, O., Marchini, J., and Zagury, J.F. (2012). A linear complexity phasing method for thousands of genomes. Nat. Methods *9*, 179–181.
32. Marchini, J., Howie, B., Myers, S., McVean, G., and Donnelly, P. (2007). A new multipoint method for genome-wide association studies by imputation of genotypes. Nat. Genet. *39*, 906–913.
33. Abecasis, G.R., Auton, A., Brooks, L.D., DePristo, M.A., Durbin, R.M., Handsaker, R.E., Kang, H.M., Marth, G.T., and McVean, G.A.; 1000 Genomes Project Consortium (2012). An integrated map of genetic variation from 1,092 human genomes. Nature *491*, 56–65.
34. Dilthey, A.T., Moutsianas, L., Leslie, S., and McVean, G. (2011). HLA*IMP—an integrated framework for imputing classical HLA alleles from SNP genotypes. Bioinformatics *27*, 968–972.
35. Leslie, S., Donnelly, P., and McVean, G. (2008). A statistical method for predicting classical HLA alleles from SNP data. Am. J. Hum. Genet. *82*, 48–56.
36. Bhattacharjee, S., Rajaraman, P., Jacobs, K.B., Wheeler, W.A., Melin, B.S., Hartge, P., Yeager, M., Chung, C.C., Chanock, S.J., and Chatterjee, N.; GliomaScan Consortium (2012). A subset-based approach improves power and interpretation for the combined analysis of genetic association studies of heterogeneous traits. Am. J. Hum. Genet. *90*, 821–835.
37. Morris, A.P. (2011). Transethnic meta-analysis of genomewide association studies. Genet. Epidemiol. *35*, 809–822.
38. Willer, C.J., Li, Y., and Abecasis, G.R. (2010). METAL: fast and efficient meta-analysis of genomewide association scans. Bioinformatics *26*, 2190–2191.
39. Wakefield, J. (2009). Bayes factors for genome-wide association studies: comparison with P-values. Genet. Epidemiol. *33*, 79–86.
40. Liu, Y., Helms, C., Liao, W., Zaba, L.C., Duan, S., Gardner, J., Wise, C., Miner, A., Malloy, M.J., Pullinger, C.R., et al. (2008). A genome-wide association study of psoriasis and psoriatic arthritis identifies new disease loci. PLoS Genet. *4*, e1000041.
41. Esparza-Gordillo, J., Weidinger, S., Fölster-Holst, R., Bauerfeind, A., Ruschendorf, F., Patone, G., Rohde, K., Marenholz, I., Schulz, F., Kerscher, T., et al. (2009). A common variant on chromosome 11q13 is associated with atopic dermatitis. Nat. Genet. *41*, 596–601.
42. Sun, L.D., Xiao, F.L., Li, Y., Zhou, W.M., Tang, H.Y., Tang, X.F., Zhang, H., Schaarschmidt, H., Zuo, X.B., Foelster-Holst, R., et al. (2011). Genome-wide association study identifies two new susceptibility loci for atopic dermatitis in the Chinese Han population. Nat. Genet. *43*, 690–694.
43. Marenholz, I., Rivera, V.A., Esparza-Gordillo, J., Bauerfeind, A., Lee-Kirsch, M.A., Ciechanowicz, A., Kurek, M., Piskackova, T., Macek, M., and Lee, Y.A. (2011). Association screening in the Epidermal Differentiation Complex (EDC) identifies an SPRR3 repeat number variant as a risk factor for eczema. J. Invest. Dermatol. *131*, 1644–1649.
44. Ellinghaus, E., Ellinghaus, D., Stuart, P.E., Nair, R.P., Debrus, S., Raelson, J.V., Belouchi, M., Fournier, H., Reinhard, C., Ding, J., et al. (2010). Genome-wide association study identifies a psoriasis susceptibility locus at TRAF3IP2. Nat. Genet. *42*, 991–995.
45. Hüffmeier, U., Uebe, S., Ekici, A.B., Bowes, J., Giardina, E., Korendowych, E., Juneblad, K., Apel, M., McManus, R., Ho, P., et al. (2010). Common variants at TRAF3IP2 are associated with susceptibility to psoriatic arthritis and psoriasis. Nat. Genet. *42*, 996–999.
46. Patel, C.V., Handy, I., Goldsmith, T., and Patel, R.C. (2000). PACT, a stress-modulated cellular activator of interferon-induced double-stranded RNA-activated protein kinase, PKR. J. Biol. Chem. *275*, 37993–37998.

47. Kok, K.H., Lui, P.Y., Ng, M.H., Siu, K.L., Au, S.W., and Jin, D.Y. (2011). The double-stranded RNA-binding protein PACT functions as a cellular activator of RIG-I to facilitate innate antiviral response. Cell Host Microbe *9*, 299–309.
48. Guttman-Yassky, E., Nograles, K.E., and Krueger, J.G. (2011). Contrasting pathogenesis of atopic dermatitis and psoriasis—part I: clinical and pathologic concepts. J. Allergy Clin. Immunol. *127*, 1110–1118.
49. Pruim, R.J., Welch, R.P., Sanna, S., Teslovich, T.M., Chines, P.S., Gliedt, T.P., Boehnke, M., Abecasis, G.R., and Willer, C.J. (2010). LocusZoom: regional visualization of genome-wide association scan results. Bioinformatics *26*, 2336–2337.
50. Elder, J.T., Bruce, A.T., Gudjonsson, J.E., Johnston, A., Stuart, P.E., Tejasvi, T., Voorhees, J.J., Abecasis, G.R., and Nair, R.P. (2010). Molecular dissection of psoriasis: integrating genetics and biology. J. Invest. Dermatol. *130*, 1213–1226.
51. Henry, J., Hsu, C.Y., Haftek, M., Nachat, R., de Koning, H.D., Gardinal-Galera, I., Hitomi, K., Balica, S., Jean-Decoster, C., Schmitt, A.M., et al. (2011). Hornerin is a component of the epidermal cornified cell envelopes. FASEB J. *25*, 1567–1576.
52. Guttman-Yassky, E., Suárez-Fariñas, M., Chiricozzi, A., Nograles, K.E., Shemer, A., Fuentes-Duculan, J., Cardinale, I., Lin, P., Bergman, R., Bowcock, A.M., and Krueger, J.G. (2009). Broad defects in epidermal cornification in atopic dermatitis identified through genomic analysis. J. Allergy Clin. Immunol. *124*, 1235–1244, e58.
53. Wu, Z., Hansmann, B., Meyer-Hoffert, U., Gläser, R., and Schröder, J.M. (2009). Molecular identification and expression analysis of filaggrin-2, a member of the S100 fused-type protein family. PLoS ONE *4*, e5227.
54. Takaishi, M., Makino, T., Morohashi, M., and Huh, N.H. (2005). Identification of human hornerin and its expression in regenerating and psoriatic skin. J. Biol. Chem. *280*, 4696–4703.
55. Kim, B.E., Howell, M.D., Guttman-Yassky, E., Gilleaudeau, P.M., Cardinale, I.R., Boguniewicz, M., Krueger, J.G., and Leung, D.Y. (2011). TNF-α downregulates filaggrin and loricrin through c-Jun N-terminal kinase: role for TNF-α antagonists to improve skin barrier. J. Invest. Dermatol. *131*, 1272–1279.
56. Wang, K., Baldassano, R., Zhang, H., Qu, H.Q., Imielinski, M., Kugathasan, S., Annese, V., Dubinsky, M., Rotter, J.I., Russell, R.K., et al. (2010). Comparative genetic analysis of inflammatory bowel disease and type 1 diabetes implicates multiple loci with opposite effects. Hum. Mol. Genet. *19*, 2059–2067.
57. de Guzman Strong, C., Conlan, S., Deming, C.B., Cheng, J., Sears, K.E., and Segre, J.A. (2010). A milieu of regulatory elements in the epidermal differentiation complex syntenic block: implications for atopic dermatitis and psoriasis. Hum. Mol. Genet. *19*, 1453–1460.
58. Faghihi, M.A., and Wahlestedt, C. (2009). Regulatory roles of natural antisense transcripts. Nat. Rev. Mol. Cell Biol. *10*, 637–643.
59. Kretz, M., Siprashvili, Z., Chu, C., Webster, D.E., Zehnder, A., Qu, K., Lee, C.S., Flockhart, R.J., Groff, A.F., Chow, J., et al. (2013). Control of somatic tissue differentiation by the long non-coding RNA TINCR. Nature *493*, 231–235.
60. Lee, G.R., Spilianakis, C.G., and Flavell, R.A. (2005). Hypersensitive site 7 of the TH2 locus control region is essential for expressing TH2 cytokine genes and for long-range intrachromosomal interactions. Nat. Immunol. *6*, 42–48.
61. Knight, J., Spain, S.L., Capon, F., Hayday, A., Nestle, F.O., Clop, A., Barker, J.N., Weale, M.E., and Trembath, R.C.; Wellcome Trust Case Control Consortium; Genetic Analysis of Psoriasis Consortium; I-chip for Psoriasis Consortium (2012). Conditional analysis identifies three novel major histocompatibility complex loci associated with psoriasis. Hum. Mol. Genet. *21*, 5185–5192.
62. Gunderson, A.J., Mohammed, J., Horvath, F.J., Podolsky, M.A., Anderson, C.R., and Glick, A.B. (2013). CD8(+) T cells mediate RAS-induced psoriasis-like skin inflammation through IFN-γ. J. Invest. Dermatol. *133*, 955–963.
63. Hijnen, D., Knol, E.F., Gent, Y.Y., Giovannone, B., Beijn, S.J., Kupper, T.S., Bruijnzeel-Koomen, C.A., and Clark, R.A. (2013). CD8(+) T cells in the lesional skin of atopic dermatitis and psoriasis patients are an important source of IFN-γ, IL-13, IL-17, and IL-22. J. Invest. Dermatol. *133*, 973–979.
64. Mangalam, A.K., Taneja, V., and David, C.S. (2013). HLA class II molecules influence susceptibility versus protection in inflammatory diseases by determining the cytokine profile. J. Immunol. *190*, 513–518.
65. Okada, Y., Han, B., Tsoi, L.C., Stuart, P.E., Ellinghaus, E., Tejasvi, T., Chandran, V., Pellett, F., Pollock, R., Bowcock, A.M., et al. (2014). Fine mapping major histocompatibility complex associations in psoriasis and its clinical subtypes. Am. J. Hum. Genet. *95*, 162–172.
66. Kapila, S., Hong, E., and Fischer, G. (2012). A comparative study of childhood psoriasis and atopic dermatitis and greater understanding of the overlapping condition, psoriasis-dermatitis. Australas. J. Dermatol. *53*, 98–105.

The American Journal of Human Genetics
Supplemental Data

Genome-wide Comparative Analysis of Atopic Dermatitis and Psoriasis Gives Insight into Opposing Genetic Mechanisms

Hansjörg Baurecht, Melanie Hotze, Stephan Brand, Carsten Büning, Paul Cormican, Aiden Corvin, David Ellinghaus, Eva Ellinghaus, Jorge Esparza-Gordillo, Regina Fölster-Holst, Andre Franke, Christian Gieger, Norbert Hubner, Thomas Illig, Alan D. Irvine, Michael Kabesch, Young A.E. Lee, Wolfgang Lieb, Ingo Marenholz, W.H. Irwin McLean, Derek W. Morris, Ulrich Mrowietz, Rajan Nair, Markus M. Nöthen, Natalija Novak, Grainne M. O'Regan, the PAGE Consortium, Stefan Schreiber, Catherine Smith, Konstantin Strauch, Philip E. Stuart, Richard Trembath, Lam C. Tsoi, Michael Weichenthal, Jonathan Barker, James T. Elder, Stephan Weidinger, Heather J. Cordell, and Sara J. Brown

Supplemental Consortia Information

Membership of the PAGE (Population Architecture using Genomics and Epidemiology) consortium

University of Michigan (UMich)

Trilokraj Tejasvi,[1] Johann E. Gudjonsson,[1] John J Voorhees,[1] Jun Ding,[2] Yanming Li,[2] Hyun M Kang,[2] Goncalo R Abecasis[2]

1 Department of Dermatology, University of Michigan Ann Arbor, MI 48109, USA

2 Department of Biostatistics, Center for Statistical Genetics, University of Michigan Ann Arbor, MI 48109, USA

University of Toronto (UToronto)

Dafna D Gladman,[3] Fawnda J Pellett,[3] Vinod Chandran,[3] Cheryl F Rosen[4]

3 Department of Medicine, Division of Rheumatology, University of Toronto, Toronto Western Hospital, Toronto, Ontario M5T 2S8, Canada

4 Department of Medicine, Division of Dermatology, University of Toronto, Toronto Western Hospital, Toronto, Ontario MST2S8, Canada

Memorial University (MU)

Proton Rahman[5]

5 Department of Medicine, Memorial University, St. John's, Newfoundland A1C 5B8, Canada

University of Tartu (UTartu) and Estonian Genome Center Univeristy of Tartu (EGCUT)

Sulev Koks,[6] Külli Kingo,[7] Tonu Esko,[8] Andres Metspalu[8]

6 Department of Physiology, Centre of Translational Medicine and Centre for Translational Genomics, University of Tartu, 50409 Tartu, Estonia

7 Department of Dermatology and Venerology, University of Tartu, 50409 Tartu, Estonia

8 Estonian Genome Center and Center of Translational Genomics; Estonian Biocenter; Institute of Molecular and Cell Biology, University of Tartu, 50409 Tartu, Estonia

The Feinstein Institute for Medical Research (FIMR)

Peter Gregersen[9]

9 Robert S. Boas Center for Genomics and Human Genetics, The Feinstein Institute for Medical Research, Manhasset, NY 11030

National Psoriasis Victor Henschel BioBank (NPF)

Andrew Henschel,[10] Marin Aurand,[10] Bruce Bebo[10]

10 National Psoriasis Foundation, Portland, OR 97223 USA;

Henry Ford Hospital (HFH)

Henry W Lim[11]

11 Henry Ford Hospital, Detroit, Michigan, 48202, USA

Supplemental Figures

Figure S1. CCMA calibration curve

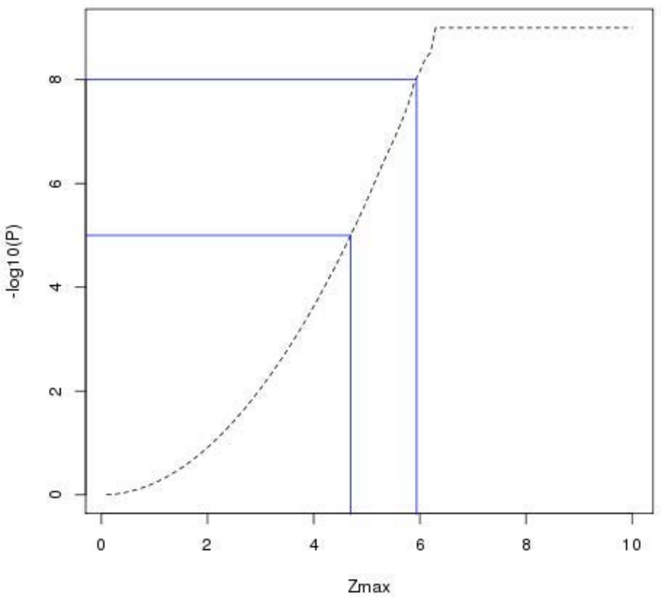

Calibration curve of the Z_{max} statistic based on 10^9 simulation replicates. Thresholds of Z_{max}=4.7 and Z_{max}=6.0 correspond to approximate genome-wide "suggestive" and "significant" thresholds (P=1x10^{-5} and 1x10^{-8}) respectively.

Figure S2. Mirrored Manhattan Plot of CCMA (upper panel) and MANTRA analysis (lower panel)

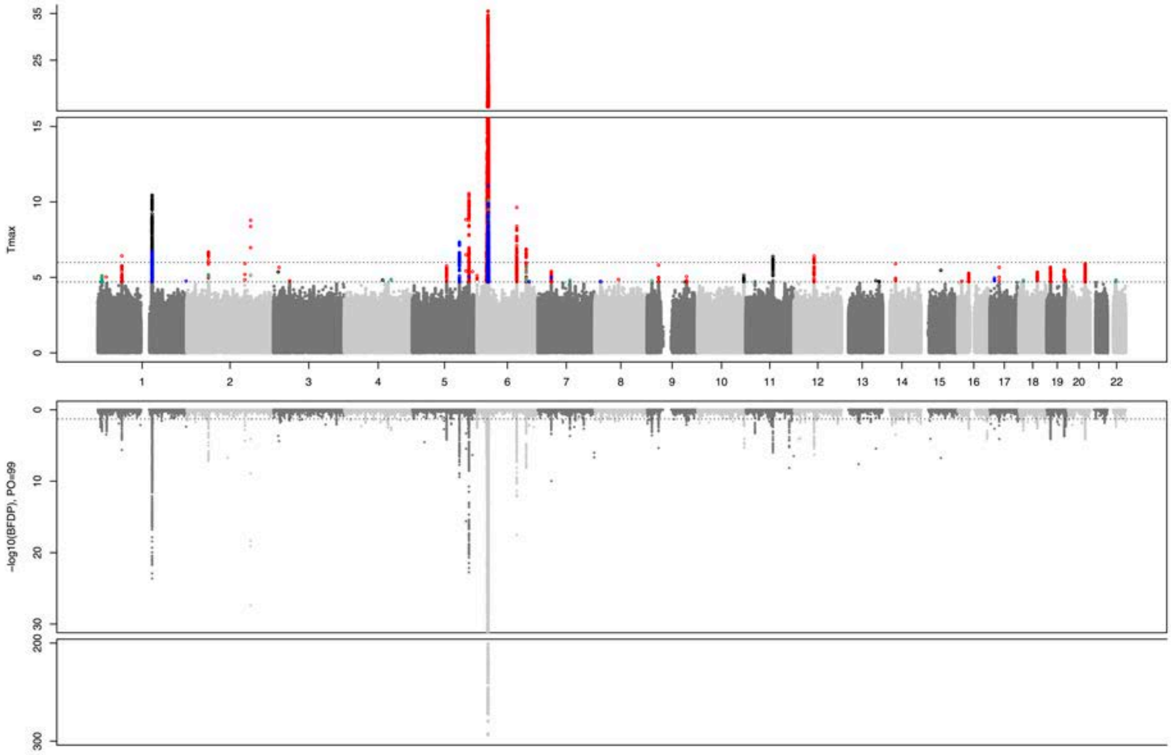

In the case-control meta-analysis (CCMA) the SNVs are color-coded as follows: **red** indicates psoriasis-specific; **black** AD-specific; **blue** opposing effect; **green** shared effect; the genome-wide significance threshold is indicated at T_{max}=6.0 (suggestive significance at T_{max}=4.7) for CCMA and Bayesian False Discovery Probability BFDP=0.05 for MANTRA.

Figure S3. Agreement of CCMA and MNM effect classification

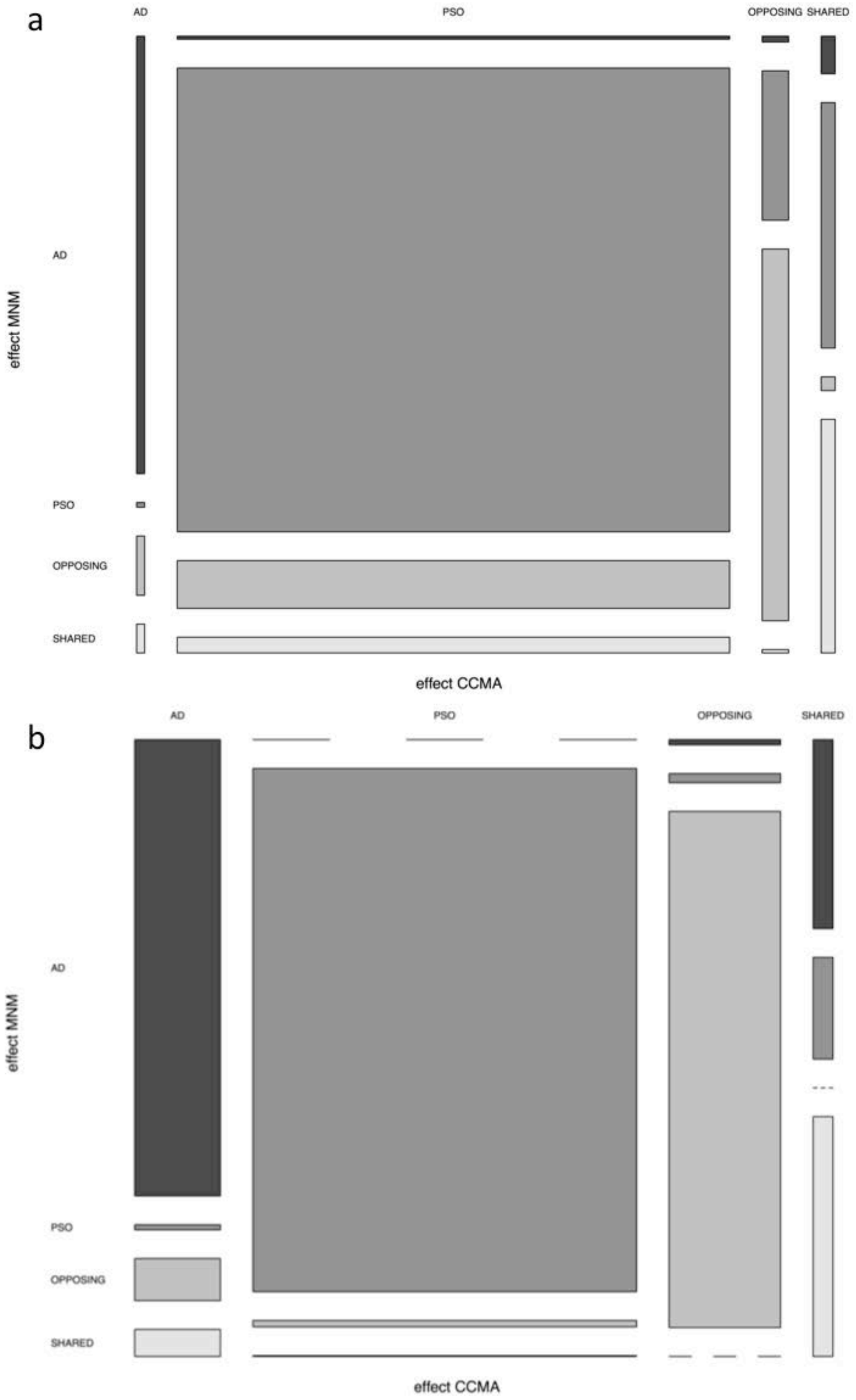

Mosaic plot showing the agreement of SNV effect classification into AD, psoriasis (PSO), shared and opposing effects for filtered SNVs (T_{max}>4.7) using CCMA and the multinomial models (MNM); (a) genome-wide comparison of both methods revealed 85.6% agreement, and (b) excluding the complex HLA region the comparison showed 94.8% agreement.

Figure S4: Association Plot and LD Structure of the EDC (left) and 5q31.1 (right)

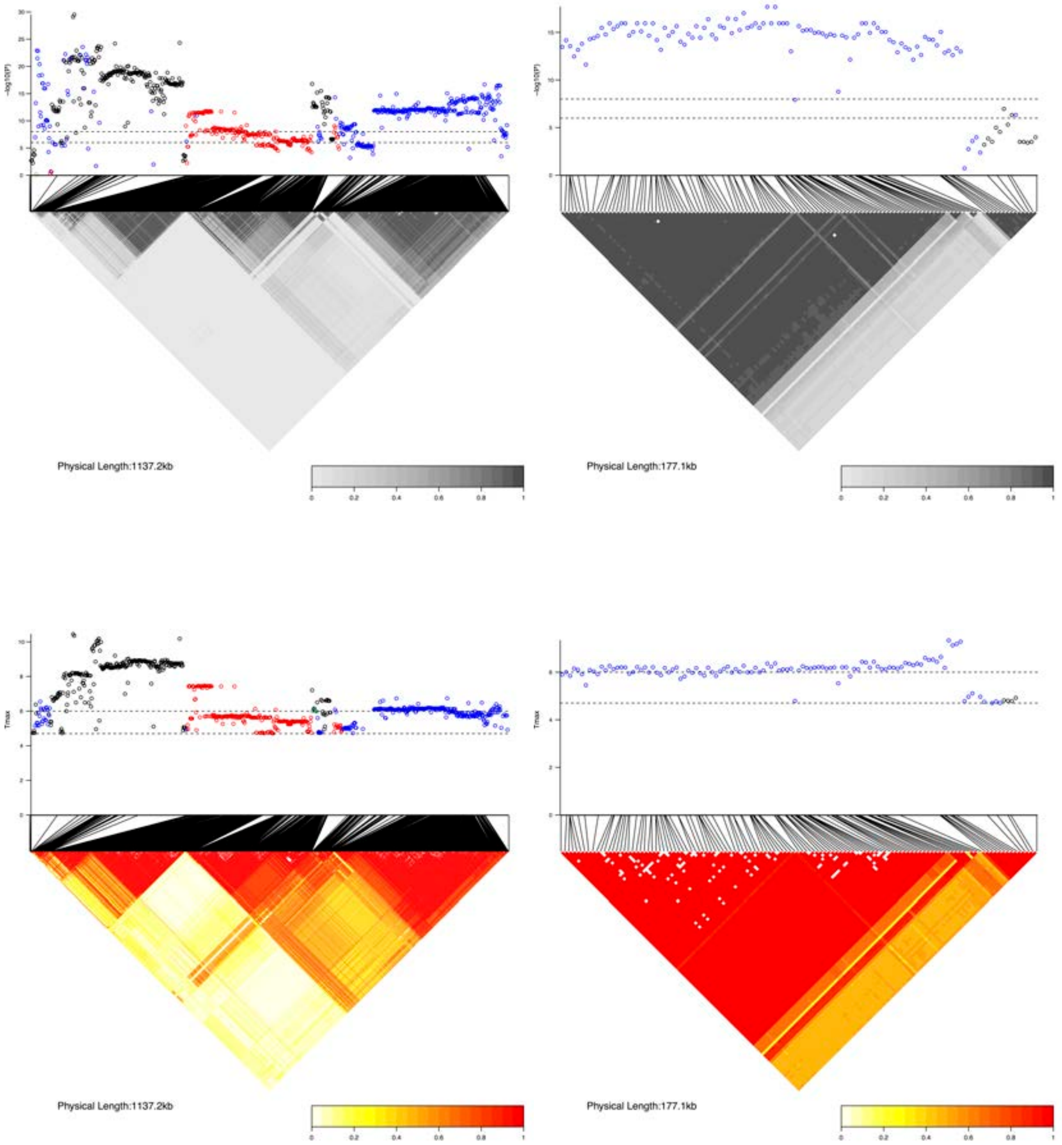

The upper plots display -$\log_{10}$(P) from the multinomial regression model and R^2 LD values; the lower plots display T_{max} from the CCMA and D' LD values; dashed lines indicate suggestive and genome-wide significance thresholds; color codes: **red**=psoriasis, **black**=AD, and **blue**=opposing effect.

Figure S5: Association Plot and LD Structure of the cytokine cluster at 5q31.1 using Immunochip data

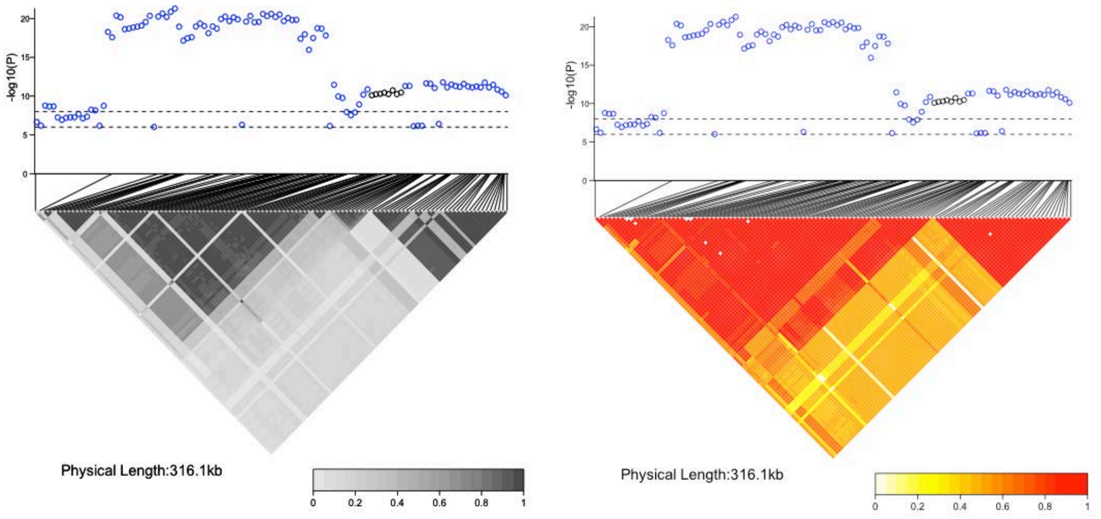

R^2 (left) and D' (right) together with $-\log_{10}(P)$ from the multinomial regression model are displayed; color codes: **black**=AD-specific effect and **blue**=opposing effect on AD and psoriasis.

Figure S6: Predicted protein network and gene ontology analyses

(a) 32 protein products & interactions predicted from 33 genes showing significance in genome-wide and/or conditional analyses

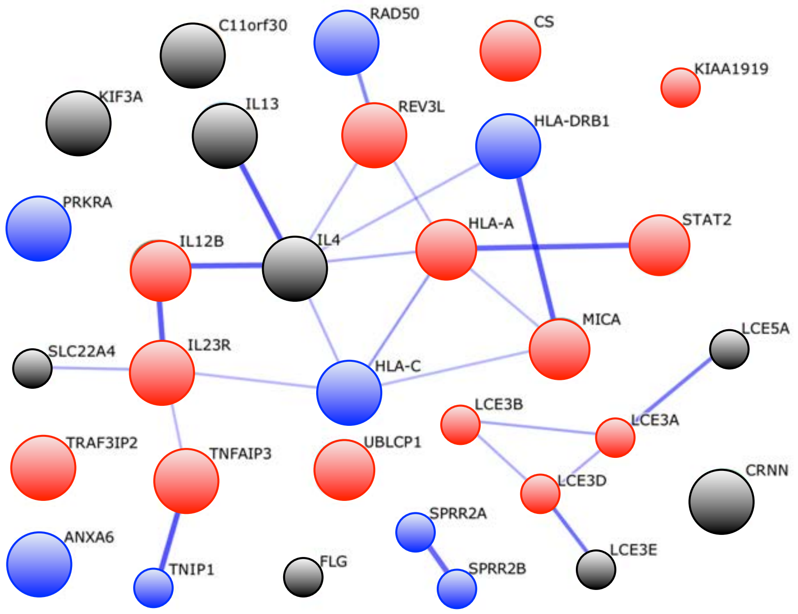

Colors indicate **AD-specific,** **psoriasis-specific** and **AD-psoriasis opposing** effects

GO:0031424	keratinization	n=7	p=3.08E-08
GO:0030216	keratinocyte differentiation	n=8	p=3.08E-08
GO:0009913	epidermal cell differentiation	n=8	p=2.64E-07
GO:0008544	epidermis development	n=8	p=4.90E-05
GO:0043588	skin development	n=8	p=1.11E-04
GO:2000319	regulation of T-helper 17 cell differentiation	n=3	p=1.50E-04
GO:2000316	regulation of T-helper 17 type immune response	n=3	p=2.57E-04

(b) 17 protein products & interactions predicted from 18 genes showing significant association with AD (AD-specific and opposing)

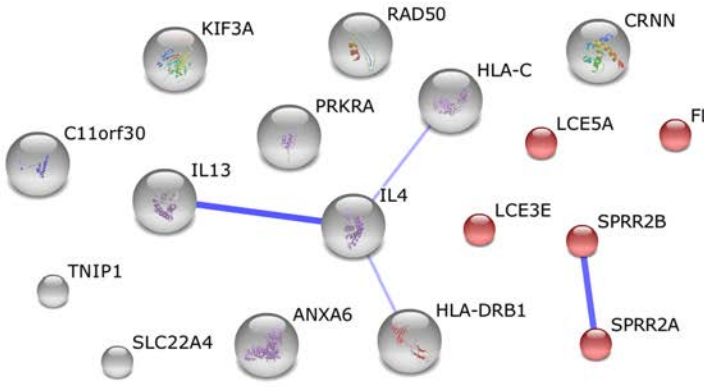

GO:0030216	**keratinocyte differentiation**	**n=5 marked red**	p=4.30E-04
GO:0031424	keratinization	n=4	p=6.94E-04
GO:0009913	epidermal cell differentiation	n=5	p=6.94E-04
GO:0008544	epidermis development	n=5	p=1.60E-02
GO:0043588	skin development	n=5	p=2.46E-02

(c) 23 protein products & interactions predicted from 24 genes showing significant association with psoriasis (psoriasis-specific and opposing)

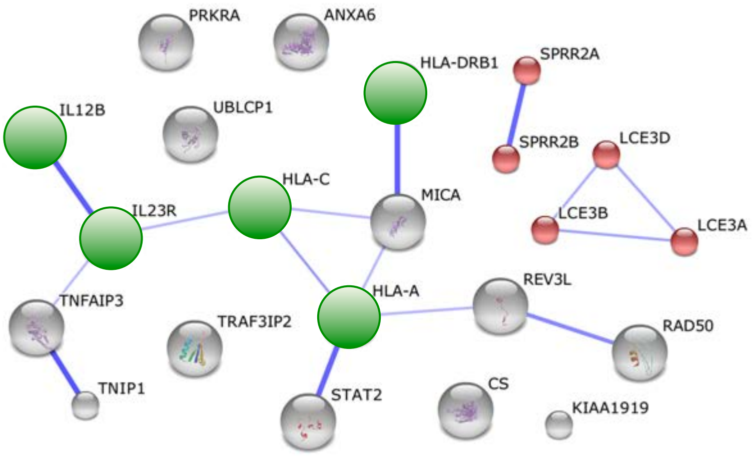

GO:0031424	**keratinization**	**n=5 marked red**	p=4.77E-05
GO:0030216	**keratinocyte differentiation**	**n=5 marked red**	p=6.94E-04
GO:0009913	epidermal cell differentiation	n=5	p=1.89E-03
GO:0034341	**response to interferon-gamma**	**n=5 marked green**	p=1.89E-03
GO:0050688	regulation of defense response to virus	n=4	p=8.11E-03

(d) 9 protein products from 12 genes showing opposing actions in AD and psoriasis

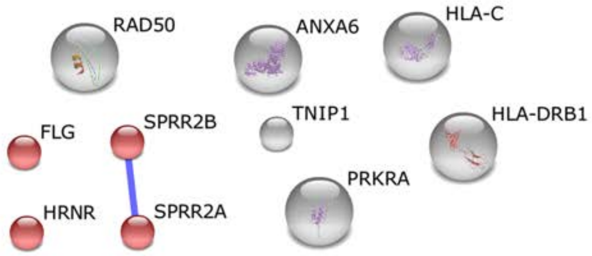

GO:0030216	**keratinocyte differentiation**	**n=4 marked red**	p=2.70E-03
GO:0009913	**epidermal cell differentiation**	**n=4 marked red**	p=4.78E-03
GO:0031424	keratinization	n=3	p=1.24E-02
GO:0061436	establishment of skin barrier	n=2	p=3.02E-02
GO:0008544	epidermis development	n=4	p=3.02E-02
GO:0033561	regulation of water loss via skin	n=2	p=4.55E-02

Genes identified from the results of analyses presented in Tables 1, 2 and 3; STRING$_{9.1}$ http://string-db.org/ accessed 17 July 2014; these are confidence views in which stronger associations are represented by thicker lines; n=number of genes; p values are corrected by FDR.

Supplemental Tables

Table S1. Case and control collections for AD and psoriasis

Panel	Numbers		Collection	Platform	Previous publications
**GWAS data**					
A	AD	663	Munich/ Bonn	Illumina 300k	[1]
Germany	Controls	786	PopGen/ ISAAC		
B	AD	993	Munich/ Kiel/ Berlin	Affymetrix 6.0	[2]
Germany	Controls	1513	KORA		
C	AD	606	Dublin/ Dundee	Illumina 610k	[1; 3]
Ireland	Controls	1794	TRINITY (Dublin)	Affymetrix 6.0	
D	Psoriasis	492	Kiel	Illlumina 550k	[4]
Germany	Controls	1161	PopGen/ KORA		
E	Psoriasis	2622	WTCCC2	Illumina 1M	[5-7]
United Kingdom	Controls	5667			
F	Psoriasis	1375	CASP	Perlegen	[8]
United States	Controls	1412			
**Immunochip data**					
Germany	AD	2425	Munich/ Bonn/ Berlin	Immunochip	[3; 5]
	Psoriasis	572	Kiel		
	Controls	5449	PopGen/ KORA/ HNR/ Munich/ Berlin/ Bonn		
United States	Psoriasis	1351	UMich/NPH/HFH		
	Controls	2694	UMich/FIMR/NPH		
Canada	Psoriasis	362	UToronto/MU		
	Controls	20	UToronto		
Estonia	Psoriasis	1295	UTartu/ EGCUT		
	Controls	898	EGCUT		

PopGen, PopGen biorepository; ISAAC, International Study of Asthma and Allergies in Childhood; TRINITY, Trinity Biobank Controls, Dublin, Ireland; WTCCC2, Wellcome Trust Case Control Consortium 2; HNR, Heinz Nixdorf Recall study; CASP, Collaborative Association Study of Psoriasis; UMich, University of Michigan; HFH, Henry Ford Hospital, NPF, National Psoriasis Foundation Victor Henschel BioBank; FIMR, The Feinstein Institute for Medical Research; UToronto, University of Toronto; MU, Memorial Hospital Newfoundland; UTartu, University of Tartu; EGCUT, Estonian Genome Center University of Tartu.

Table S2. Summary data before imputation

Data set	# Individuals	Cases	Controls	# SNPs before QC	# SNPs after QC
German AD Illumina	1,381	615	766	275,099	264,658
German AD Affymetrix	2,196	892	1,304	783,889	481,680
Irish AD Illumina/Affymetrix	2,369	572	1,797	143,001	131,629
German Psoriasis	1,598	475	1,123	561,466	493,992
UK Psoriasis	7,893	2,382	5,511	542,011	508,084
US Psoriasis	2,753	1,355	1,398	599,164	327,097
	18,190	6,291	11,834		

Table S3. Summary data after imputation

Cohort	#SNPs after imputation	# SNPs failed imputation QC	# SNPs after imputation QC
German AD Illumina	5,513,905	84,450	5,429,455
German AD Affymetrix	5,552,779	75,913	5,476,866
Irish AD Illumina/Affymetrix	5,489,685	353,166	5,136,519
German Psoriasis	5,559,007	76,170	5,482,837
UK Psoriasis	5,562,502	69,402	5,493,100
US Psoriasis	5,534,690	79,330	5,455,360

Table S4. Components of the prior matrix for MANTRA

$D_{Ethnicity}$

	gerA	gerI	Ire	gerP	UK	US
gerA	0					
gerI	0.0021	0				
Ire	0.0194	0.0194	0			
gerP	0.0033	0.0024	0.0191	0		
UK	0.0109	0.0115	0.0105	0.0107	0	
US	0.0061	0.0072	0.0143	0.0072	0.0053	0

$D_{Disease}$

	gerA	gerI	Ire	gerP	UK	US
gerA	0					
gerI	0	0				
Ire	0.02	0.02	0			
gerP	0.04	0.04	0.04	0		
UK	0.04	0.04	0.04	0	0	
US	0.04	0.04	0.04	0	0	0

D_{Total}

	gerA	gerI	Ire	gerP	UK	US
gerA	0					
gerI	0.0021	0				
Ire	0.0394	0.0394	0			
gerP	0.0433	0.0424	0.0591	0		
UK	0.0509	0.0515	0.0505	0.0107	0	
US	0.0461	0.0472	0.0543	0.0072	0.0053	0

The disease prior for the MANTRA method was specified by two components (a) $D_{Ethnicity}$ showing the Euclidean distances between study centers based on the genetic distance derived by the MDS analysis of the IBS matrix and (b) $D_{Disease}$ an arbitrary distance set to distinguish both diseases (see **Methods** section). gerA: German Affymentrix AD cohort (Panel B); gerA: German Illumina AD cohort (Panel A); Ire: Irish AD cohort (Panel C); gerP: German psoriasis cohort (Panel D); UK: UK psoriasis cohort (Panel E); US: US psoriasis cohort (Panel F).

Table S5. Results of CCMA and MNM analyses showing significance levels and comparison with known susceptibility loci for AD and psoriasis

[Supplemental Table 5 provided as Excel file]

Lead SNP defined by clumping procedure (Purcell, S. et al. PLINK: a tool set for whole-genome association and population-based linkage analyses. Am J Hum Genet 81, 559-75 2007); genome-wide significant results defined as Tmax>6, BFDP<0.05 with PO=1/999 or PMNM<10^{-8} are marked in bold; genes and transcripts identified from UCSC Genome Browser Human Feb. 2009 (GRCh37/hg19) Assembly accessed 21 March 2014.

Table S6. Summary of previous GWAS findings and comparison with our findings

S6(a) Atopic Dermatitis

Locus	Reported gene(s)	Reported SNV(s)	Associated SNV in region (+/-250kb)	Position	EA / RA	OR (95% CI)	P-value	Reference	SNV represented in disease-specific/ comparative analyses?
1q21.3	*tags FLG signal*	rs3126085	rs11205006	152440176	A/T	1.615(1.543-1.691)	1.57E-25	[2; 9]	913/280
2q12.1	*IL18R1, IL18RAP ,SLC9A4*	rs13015714[a] rs759382	rs759382	103094213	T/G	0.857(0.815-0.9)	0.0017	[3; 10]	1361/0
4q27	*IL2, IL21*	rs17389644	rs17389644	123497697	A/G	1.208(1.119–1.304)	1.16E−06	[3]	550/13
5q31.1	*RAD50, IL13, IL4, KIF3A*	rs1295686[a] rs2897442	rs1295686	131995843	T/C	1.347(1.28-1.418)	5.30E-09	[10; 11]	765/121
6p21.33	*HLA-C, HLA-B, MICA*	rs9368677[a] rs2251396	rs148203517	31324100	T/G	1.305(1.239-1.375)	2.95E-07	[1; 10]	8172/6065
6p21.33	*BAT1*	rs2844509	rs2844509	31510924	A/G	1.211(1.097-1.337)	1.50E-04	[1]	5429/3596
6p21.33	*C6orf48*	rs9368699	rs9368699	31802541	C/T	0.657(0.534-0.809)	7.78E-05	[1]	719/366
6p21.33	*TNXB, CREBL1*	rs12153855	rs12153855	32074804	C/T	0.781(0.679-0.898)	5.19E-04	[1]	1755/1027
11p12	*PRR5L*	rs12295535	rs12295535	36432024	T/C	1.382(1.202-1.589)	0.0204	[3]	1141/1
11q13.1	*OVOL1*	rs479844	rs479844	65551957	A/G	0.851(0.815-0.889)	2.06E-04	[10,11]	838/0
11q13.5	*C11orf30, LRRC32*	rs7927894, rs11236809[a]	rs7927894	76301316	T/C	1.265(1.21-1.322)	1.24E-07	[2; 10]	1104/48
16p13.13	*CLEC16A*	rs9923856[a] rs2041733	rs2041733	11229589	T/C	1.172(1.124-1.222)	1.51E-04	[3; 10]	1292/2
17q21.32 -33	*ZNF652*	rs16948048	rs16948048	47440466	G/A	1.101(1.012-1.198)	0.0252	[3]	991/0
19p13.2	*ACTL9*	rs2164983	rs2164983	8789381	A/C	1.129(0.999-1.275)	0.0521	[11]	1002/0
20q13.33	*TNFRSF6B*	rs909341	rs909341	62328742	G/A	1.316(1.205-1.429)	7.73E−10	[3]	1226/0
**Loci reported in Asian populations**									
3p22.3	*GLB1*	rs7613051	rs1607463	32910192	A/T	1.471(1.294-1.673)	0.0027	[10]	1032/0
3q13.2	*CCDC80*	rs12634229	rs58161637	112545910	G/GT	1.214(1.16-1.27)	2.21E-05	[10]	1038/0
5q22.1	*TMEM232, SLC25A46*	rs7701890	rs4957919	110100063	T/C	1.195(1.117-1.28)	0.0089	[9]	909/0
6p21.32	*GPSM3*	rs176095	rs176095	32158319	G/A	0.768(0.694-0.85)	3.44E-07	[10]	2622/1554
6p21.32	*C6orf10*	rs9469099	rs41268896	32070069	A/G	1.298(1.238-1.361)	4.16E-08	[10]	8274/5295
7p22.2	*CARD11*	rs4722404	rs6953573	3081727	A/G	1.194(1.136-1.255)	3.61E-04	[10]	1813/0
10q21.2	*ZNF365*	rs10995251	rs2393903	64380336	T/C	0.831(0.795-0.869)	3.42E-05	[10]	951/0
11p15.4	*OR10A3, NLRP10*	rs878860	rs4758289	8115699	T/C	1.163(1.099-1.231)	0.0079	[10]	1752/0
20q13.2	*CYP24A1, PFDN4*	rs16999165	rs176383	52613413	T/C	1.356(1.253-1.468)	1.19E-04	[10]	1286/0

EA/RA=effect allele/reference allele; [a]indicate reported Asian SNVs.

S6(b) Psoriasis

Locus	Reported gene(s)	Reported SNV(s)	Associated SNV in region (+/-250kb)	Position	EA / RA	OR (95% CI)	P-value	Reference	SNV represented in disease-specific/ comparative analyses?
1p36.23	*SLC45A1, TNFRSF9*	rs11121129	rs11121129	8268095	A/G	1.131(1.096-1.167)	8.74E-05	5	884/25
1p36.13	*CAPZB*	rs7667	rs7667	19718824	A/G	1.079(1.046-1.112)	0.0131	4	1127/0
1p36.11	*IL28RA*	rs4649203	rs4649203	24519920	A/G	1.153(1.113-1.195)	5.81E-05	5; 7	973/2
1p36.11	*RUNX3*	rs7536201	rs7536201	25293084	T/C	0.883(0.858-0.91)	2.37E-05	5	1051/50
1p31.3	*IL23R*	rs2201841,<br>rs11209026<br>rs9988642	rs11209026	67705958	A/G	0.646(0.598-0.699)	2.90E-08	5; 7; 8	1066/60
1q21.3	*LCE3A, LCE3B, LCE3D*	rs4085613[a]<br>rs4112788<br>rs6677595	rs4112788	152551276	A/G	0.791(0.766-0.816)	8.37E-14	5; 7; 12	1028/492
2p16.1	*FLJ16341, REL*	rs702873,<br>rs62149416	rs702873	61081542	T/C	0.822(0.798-0.847)	3.22E-11	5; 7; 13	649/64
2p15	*B3GNT2*	rs10865331	rs10865331	62551472	A/G	1.131(1.067-1.198)	3.43E-05	5	836/0
2q24.2	*IFIH1*	rs17716942	rs17716942	163260691	T/C	1.309(1.198-1.433)	3.26E-09	7	459/3
3p24.3	*Intergenic*	rs6809854	rs6809854	18784423	A/G	0.885(0.854-0.917)	5.73E-04	7	1126/12
3q13.31	*ZDHHC23*	rs1386478	rs1386478	113680951	A/G	1.09(1.022-1.162)	0.00894	4	711/0
5q15	*ERAP1*	rs27524,<br>rs27432	rs27432	96119273	A/G	1.195(1.157-1.234)	2.37E-08	5; 7	1199/65
5q31.1	*IL13, IL4*	rs20541,<br>rs1295685	rs20541	131995964	A/G	0.841(0.81-0.874)	5.39E-06	5; 8	766/121
5q33.1	*ANXA6, TNIP1*	rs2233278,<br>rs17728338	rs17728338	150478318	A/G	1.647(1.557-1.743)	1.07E-18	5; 8	1435/3
5q33.3	*IL12B*	rs2082412<br>rs3213094[a]<br>rs2546890<br>rs12188300	rs2546890	158759900	A/G	1.368(1.328-1.41)	5.39E-26	5; 8; 12; 14	801/218
6p25.3	*EXOC2, IRF4*	rs9504361	rs9504361	577820	A/G	1.163(1.129-1.198)	2.96E-07	5	1240/15
6p21.33	*HLA-C*	rs1265181[a]<br>rs12191877<br>rs4406273<br>rs10484554<br>rs3134792<br>rs2395029	rs2395029	31431780	G/T	5.00(4.695-5.348)	6.84E-138	5; 8; 12; 15; 16	6809/4811
6q21	*REV3L*	rs465969	rs465969	111655530	A/G	1.403(1.335-1.474)	7.82E-12	7	519/226
6q21	*TRAF3IP2*	rs240993,<br>rs33980500	rs240993	111673714	T/C	1.282(1.241-1.324)	1.20E-14	7; 14	543/235
6q23.3	*TNFAIP3*	rs582757<br>rs610604	rs582757	138197824	T/C	0.802(0.777-0.828)	5.70E-12	5; 7	921/60
6q25.3	TAGAP	rs2451258	rs2451258	159506600	T/C	0.898(0.871-0.926)	4.13E-04	5	1558/4
7p14.1-2	*ELMO1*	rs2700987	rs2700987	37386237	A/C	1.174(1.14-1.209)	6.60E-08	5	1193/27
9p21.1	*DDX58*	rs11795343	rs11795343	32523737	T/C	1.19(1.155-1.226)	5.78E-09	5	893/29
9q31.2	*KLF4*	rs10979182	rs10979182	110817020	A/G	1.149(1.084-1.218)	2.99E-06	5	1265/6
9q34.13	*TSC1*	rs1076160	rs1076160	135776034	T/C	1.046(0.988-1.107)	0.1223	8	1079/0
10q22.3	*ZMIZ1*	rs1250546<br>rs1250544	rs1250546	81032532	A/G	1.114(1.05-1.182)	3.55E-04	4; 5	1197/0
11q13.1	*RPS6KA4, PRDX5*	rs645078	rs645078	64135298	A/C	1.095(1.032-1.163)	0.0029	5	608/0
11q22.3	*ZC3H12C*	rs4561177	rs4561177	109962432	A/G	1.122(1.059-1.189)	9.03E-05	5	858/0
11.q24.3	*ETS1*	rs3802826	rs3802826	128406438	A/G	1.116(1.054-1.182)	1.67E-04	5	1010/9
12q13.2	*RPS26*	rs12580100	rs12580100	56439209	A/G	1.284(1.171-1.408)	1.01E-07	13	315/40
12q13.3	*IL23A, STAT2*	rs2066808<br>rs2066819	rs2066808	56737973	A/G	1.477(1.305-1.671)	6.45E-10	5; 8	420/32
13q14.11	*COG6*	rs7993214	rs7993214	40350912	T/C	0.897(0.87-0.925)	3.66E-04	16	1133/0
14q13.2	*NFKBIA*	rs12586317<br>rs8016947	rs8016947	35832666	T/G	0.84(0.815-0.865)	3.90E-09	7; 13	916/8
16p11.2	*FBXL19, STX1B*	rs10782001<br>rs12445568	rs12445568	31004812	C/T	1.167(1.101-1.238)	2.65E-07	5; 13	343/100

S6(b) continued

Locus	Reported gene(s)	Reported SNV(s)	Associated SNV in region (+/-250kb)	Position	EA / RA	OR (95% CI)	P-value	Reference	SNV represented in disease-specific/ comparative analyses?
16p13.13	*PRM3, SOCS1*	rs4780355	rs4780355	11347858	T/C	1.165(1.127-1.203)	3.13E-06	[4]	1655/2
17q11.2	*NOS2*	rs4795067 rs28998802	rs4795067	26106675	A/G	0.843(0.818-0.869)	1.47E-08	[5; 13]	836/3
17q21.2	*PTRF, STAT3*	rs963986	rs963986	40561579	C/G	1.158(1.069-1.255)	3.21E-04	[5]	642/1
17q25.3	*CARD14*	rs11652075	rs11652075	78178893	T/C	0.885(0.86-0.912)	3.64E-05	[5]	1347/11
18q21.2	*POL1, STARD6, MBD2*	rs545979	rs545979	51819750	T/C	1.172(1.136-1.209)	3.29E-07	[5]	736/84
19p13.2	*TYK2*	rs34536443 rs12720356 rs280519	rs34536443	10463118	G/C	1.76(1.46-2.10)	3.4E-10	[5; 7]	902/29
19p13.2	*ILF3, CARM1*	rs892085	rs892085	10818092	A/G	1.172(1.103-1.245)	3.13E-07	[5]	810/42
20q13.12	*SDC4*	rs1008953	rs1008953	43980726	C/T	1.121(1.046-1.202)	0.0013	[13]	1351/0
20q13.13	*SPATA2, RNF114*	rs495337 rs1056198	rs495337	48522330	A/G	0.839(0.814-0.864)	3.43E-09	[5; 15]	1078/215
22q11.21	*UBE2L3*	rs4821124	rs4821124	21979289	C/T	1.159(1.076-1.248)	9.08E-05	[5]	535/0
**Loci reported in Asian populations**									
4q24	*NFKB1*	rs1020760 rs1609798	rs230503	103492669	A/G	0.923(0.893-0.955)	0.0170	[17]	835/0
5q33.3	*PTTG1*	rs2431697	rs2961918	159901408	A/C	1.111(1.075-1.149)	0.0015	[18]	1039/0
8p23.2	*CSMD1*	rs7007032	rs11136687	3809504	A/T	1.127(1.087-1.168)	7.88E-04	[18]	3475/0
12p13.3	Multiple genes: *CD27-LAG3*	rs758739 rs2243750	rs111922468	6556078	T/C	1.121(1.08-1.163)	0.0020	[17]	993/0
13q12.11	*GJB2*	rs3751385	rs7324150	20698681	A/G	0.878(0.84-0.917)	0.0029	[18]	1133/0
17q12	*IKZF3*	rs10852936 rs12936231	rs146489775	38185192	T/C	1.773(1.418-2.216)	0.0103	[17]	634/0
18q22.1	*SERPINB8*	rs514315	rs191358722	61730218	T/C	1.676(1.463-1.921)	1.51E-04	[18]	1215/0
19q13.33	*FUT2*	rs1047781	rs629504	49223633	C/G	0.845(0.819-0.871)	4.14E-08	[19]	1186/27
19q13.41	*ZNF816A*	rs12459008 rs9304742	rs12459008	53454789	A/T	1.162(1.090-1.238)	3.8E-06	[18; 19]	2016/13

EA/RA=effect allele/reference allele; [a]indicate reported Asian SNVs.

Table S7. Gene expression data in psoriasis and AD relating to candidate genes identified by comparative analysis

[Supplemental Table 7 provided as Excel file]

Gene expression data available from published and publically available datasets are summarized for candidate genes.

References

1. Weidinger, S., Willis-Owen, S.A., Kamatani, Y., Baurecht, H., Morar, N., Liang, L., Edser, P., Street, T., Rodriguez, E., O'Regan, G.M., et al. (2013). A genome-wide association study of atopic dermatitis identifies loci with overlapping effects on asthma and psoriasis. Hum Mol Genet 22, 4841-4856.

2. Esparza-Gordillo, J., Weidinger, S., Folster-Holst, R., Bauerfeind, A., Ruschendorf, F., Patone, G., Rohde, K., Marenholz, I., Schulz, F., Kerscher, T., et al. (2009). A common variant on chromosome 11q13 is associated with atopic dermatitis. Nat Genet 41, 596-601.

3. Ellinghaus, D., Baurecht, H., Esparza-Gordillo, J., Rodriguez, E., Matanovic, A., Marenholz, I., Hubner, N., Schaarschmidt, H., Novak, N., Michel, S., et al. (2013). High-density genotyping study identifies four new susceptibility loci for atopic dermatitis. Nature Genetics 45, 808-812.

4. Ellinghaus, D., Ellinghaus, E., Nair, R.P., Stuart, P.E., Esko, T., Metspalu, A., Debrus, S., Raelson, J.V., Tejasvi, T., Belouchi, M., et al. (2012). Combined analysis of genome-wide association studies for Crohn disease and psoriasis identifies seven shared susceptibility loci. American Journal of Human Genetics 90, 636-647.

5. Tsoi, L.C., Spain, S.L., Knight, J., Ellinghaus, E., Stuart, P.E., Capon, F., Ding, J., Li, Y., Tejasvi, T., Gudjonsson, J.E., et al. (2012). Identification of 15 new psoriasis susceptibility loci highlights the role of innate immunity. Nature Genetics 44, 1341-1348.

6. Knight, J., Spain, S.L., Capon, F., Hayday, A., Nestle, F.O., Clop, A., Wellcome Trust Case Control, C., Genetic Analysis of Psoriasis, C., Consortium, I.c.f.P., Barker, J.N., et al. (2012). Conditional analysis identifies three novel major histocompatibility complex loci associated with psoriasis. Hum Mol Genet 21, 5185-5192.

7. Genetic Analysis of Psoriasis Consortium, Wellcome Trust Case Control Consortium, Strange, A., Capon, F., Spencer, C.C., Knight, J., Weale, M.E., Allen, M.H., Barton, A., Band, G., et al. (2010). A genome-wide association study identifies new psoriasis susceptibility loci and an interaction between HLA-C and ERAP1. Nature Genetics 42, 985-990.

8. Nair, R.P., Duffin, K.C., Helms, C., Ding, J., Stuart, P.E., Goldgar, D., Gudjonsson, J.E., Li, Y., Tejasvi, T., Feng, B.J., et al. (2009). Genome-wide scan reveals association of psoriasis with IL-23 and NF-kappaB pathways. Nature Genetics 41, 199-204.

9. Sun, L.D., Xiao, F.L., Li, Y., Zhou, W.M., Tang, H.Y., Tang, X.F., Zhang, H., Schaarschmidt, H., Zuo, X.B., Foelster-Holst, R., et al. (2011). Genome-wide association study identifies two new susceptibility loci for atopic dermatitis in the Chinese Han population. Nat Genet 43, 690-694.

10. Hirota, T., Takahashi, A., Kubo, M., Tsunoda, T., Tomita, K., Sakashita, M., Yamada, T., Fujieda, S., Tanaka, S., Doi, S., et al. (2012). Genome-wide association study identifies eight new susceptibility loci for atopic dermatitis in the Japanese population. Nat Genet 44, 1222-1226.

11. Paternoster, L., Standl, M., Chen, C.M., Ramasamy, A., Bonnelykke, K., Duijts, L., Ferreira, M.A., Alves, A.C., Thyssen, J.P., Albrecht, E., et al. (2011). Meta-analysis of genome-wide association studies identifies three new risk loci for atopic dermatitis. Nature Genetics 44, 187-192.

12. Zhang, X.J., Huang, W., Yang, S., Sun, L.D., Zhang, F.Y., Zhu, Q.X., Zhang, F.R., Zhang, C., Du, W.H., Pu, X.M., et al. (2009). Psoriasis genome-wide association study identifies susceptibility variants within LCE gene cluster at 1q21. Nature Genetics 41, 205-210.

13. Stuart, P.E., Nair, R.P., Ellinghaus, E., Ding, J., Tejasvi, T., Gudjonsson, J.E., Li, Y., Weidinger, S., Eberlein, B., Gieger, C., et al. (2010). Genome-wide association analysis identifies three psoriasis susceptibility loci. Nature Genetics 42, 1000-1004.

14. Ellinghaus, E., Ellinghaus, D., Stuart, P.E., Nair, R.P., Debrus, S., Raelson, J.V., Belouchi, M., Fournier, H., Reinhard, C., Ding, J., et al. (2010). Genome-wide association study identifies a psoriasis susceptibility locus at TRAF3IP2. Nature Genetics 42, 991-995.

15. Capon, F., Bijlmakers, M.J., Wolf, N., Quaranta, M., Huffmeier, U., Allen, M., Timms, K., Abkevich, V., Gutin, A., Smith, R., et al. (2008). Identification of ZNF313/RNF114 as a novel psoriasis susceptibility gene. Human molecular genetics 17, 1938-1945.

16. Liu, Y., Helms, C., Liao, W., Zaba, L.C., Duan, S., Gardner, J., Wise, C., Miner, A., Malloy, M.J., Pullinger, C.R., et al. (2008). A genome-wide association study of psoriasis and psoriatic arthritis identifies new disease loci. PLoS Genet 4, e1000041.

17. Sheng, Y., Jin, X., Xu, J., Gao, J., Du, X., Duan, D., Li, B., Zhao, J., Zhan, W., Tang, H., et al. (2014). Sequencing-based approach identified three new susceptibility loci for psoriasis. Nature communications 5, 4331.

18. Sun, L.D., Cheng, H., Wang, Z.X., Zhang, A.P., Wang, P.G., Xu, J.H., Zhu, Q.X., Zhou, H.S., Ellinghaus, E., Zhang, F.R., et al. (2010). Association analyses identify six new psoriasis susceptibility loci in the Chinese population. Nature genetics 42, 1005-1009.

19. Tang, H., Jin, X., Li, Y., Jiang, H., Tang, X., Yang, X., Cheng, H., Qiu, Y., Chen, G., Mei, J., et al. (2014). A large-scale screen for coding variants predisposing to psoriasis. Nature genetics 46, 45-50.
